# Supplementary material for: A Delphi process to build consensus on revised Emergency Obstetric and Newborn Care (EmONC) signal functions and levels of care
Source: PLoS One. 2025 Sep 22;20(9):e0331684. doi: 10.1371/journal.pone.0331684 (PMC12453252; doi:10.1371/journal.pone.0331684)
Supplement: S1 Appendix — (DOCX) [file pone.0331684.s001.docx]

**S1 Appendix. Delphi study invitation email**

|  |
| --- |

*Dear Colleague,*

*Due to your expertise in maternal and newborn health, we invite you to take part in an online Delphi survey on obstetric and newborn signal functions and levels of care.*

*This consultation is part of the Re-Visioning Emergency Obstetric and Newborn Care (EmONC) project; an initiative to review, rethink, and revise the*[*Emergency Obstetric care (EmOC) monitoring framework*](https://nam02.safelinks.protection.outlook.com/?url=https%3A%2F%2Fwww.who.int%2Freproductivehealth%2Fpublications%2Fmonitoring%2F9789241547734%2Fen&data=05%7C01%7Cig2449%40cumc.columbia.edu%7C962a8747c57c4e987f6e08db401091ec%7Cb0002a9b0017404d97dc3d3bab09be81%7C0%7C0%7C638174212141245233%7CUnknown%7CTWFpbGZsb3d8eyJWIjoiMC4wLjAwMDAiLCJQIjoiV2luMzIiLCJBTiI6Ik1haWwiLCJXVCI6Mn0%3D%7C3000%7C%7C%7C&sdata=DY1u8yJQUC9RT0BUYhNcsuYLv2%2BEpRRILUpmsOe0zXI%3D&reserved=0)*. The project is coordinated by the Averting Maternal Death and Disability (AMDD) program at Columbia University Mailman School of Public Health, in collaboration with UNICEF, UNFPA, WHO and the London School of Hygiene & Tropical Medicine with the aim of creating a revised obstetric and newborn care framework with indicators, tools, and guidance that meet country needs.*

*The Emergency Obstetric Care (EmOC) framework, originally published in 1997, is built around****signal functions****, categorised by two****levels of care****: basic and comprehensive. This common language for policy makers, measurement experts, clinicians and researchers has been enormously influential, but much has changed in the maternal and newborn health landscape since it was last revised in 2009.*

*Using a Delphi survey format, the purpose of this study is to build consensus on obstetric and newborn care signal functions and organisation of levels of care in low- and middle-income countries. This round of the survey will take about 20-30+ minutes to complete.  A modified survey, based on expert participants’ input in this round, will then be returned to you twice over the next 3-6 months with the goal of reaching expert consensus.*

*We would be grateful for your contribution as an expert to the Re-Visioning EmONC project by participating in this Delphi survey process. Participants who complete* *all rounds of the survey will be offered collaborative group authorship on the resulting Delphi study publication.*

*Please complete the survey here:*

*English:*[*https://www.smartsurvey.co.uk/s/Round1EmONC/*](https://nam02.safelinks.protection.outlook.com/?url=https%3A%2F%2Fwww.smartsurvey.co.uk%2Fs%2FRound1EmONC%2F&data=05%7C01%7Cig2449%40cumc.columbia.edu%7C962a8747c57c4e987f6e08db401091ec%7Cb0002a9b0017404d97dc3d3bab09be81%7C0%7C0%7C638174212141245233%7CUnknown%7CTWFpbGZsb3d8eyJWIjoiMC4wLjAwMDAiLCJQIjoiV2luMzIiLCJBTiI6Ik1haWwiLCJXVCI6Mn0%3D%7C3000%7C%7C%7C&sdata=xAkaPiRC0TyihTjYp%2F39ZftblULiBzh6nAmjA3Q1IpE%3D&reserved=0)

*French:*[*https://www.smartsurvey.co.uk/s/SONUDelphiR1FR/*](https://nam02.safelinks.protection.outlook.com/?url=https%3A%2F%2Fwww.smartsurvey.co.uk%2Fs%2FSONUDelphiR1FR%2F&data=05%7C01%7Cig2449%40cumc.columbia.edu%7C962a8747c57c4e987f6e08db401091ec%7Cb0002a9b0017404d97dc3d3bab09be81%7C0%7C0%7C638174212141401469%7CUnknown%7CTWFpbGZsb3d8eyJWIjoiMC4wLjAwMDAiLCJQIjoiV2luMzIiLCJBTiI6Ik1haWwiLCJXVCI6Mn0%3D%7C3000%7C%7C%7C&sdata=r9VuDjT2L8P%2FLk8ZRJXFeVXzMh0nQ26t2%2BkC8qK%2FLao%3D&reserved=0)

*Spanish:*[*https://www.smartsurvey.co.uk/s/AONUDelphiR1ES/*](https://nam02.safelinks.protection.outlook.com/?url=https%3A%2F%2Fwww.smartsurvey.co.uk%2Fs%2FAONUDelphiR1ES%2F&data=05%7C01%7Cig2449%40cumc.columbia.edu%7C962a8747c57c4e987f6e08db401091ec%7Cb0002a9b0017404d97dc3d3bab09be81%7C0%7C0%7C638174212141401469%7CUnknown%7CTWFpbGZsb3d8eyJWIjoiMC4wLjAwMDAiLCJQIjoiV2luMzIiLCJBTiI6Ik1haWwiLCJXVCI6Mn0%3D%7C3000%7C%7C%7C&sdata=hjg41XutD2pdWZL7ToWA9hbJoZu84Z8xvpkASosEb0o%3D&reserved=0)

*by****6^th^ December 2021.***

*If you have any questions at any time, please feel free to get in touch.*

***Thank you and best regards,***

*Dr Sarah Moxon, Dr Sudha Sharma and Dr Jalemba Aluvaala
On behalf of the Re-Visioning EmONC Steering Committee*
