# Supplementary material for: A Delphi process to build consensus on revised Emergency Obstetric and Newborn Care (EmONC) signal functions and levels of care
Source: PLoS One. 2025 Sep 22;20(9):e0331684. doi: 10.1371/journal.pone.0331684 (PMC12453252; doi:10.1371/journal.pone.0331684)
Supplement: S2 Appendix — (DOCX) [file pone.0331684.s002.docx]

**S2 Appendix. Recruitment survey**

**The Re-Visioning EmONC Delphi Registration Survey**

**Thank you for showing an interest in our Delphi study on obstetric and newborn care signal functions and levels of care!**

The 2009 WHO publication ***Monitoring emergency obstetric care: A Handbook***[1] describes a set of connected indicators to measure and monitor the availability, utilization, and quality of emergency obstetric care (EmOC) in low- and middle-income countries. This framework is built around signal functions, which are a shortlist of nine key medical interventions that can "signal" the level of functionality of a health facility. Facilities are subsequently classified as having provided either basic emergency obstetric care (BEmOC) if they performed seven of the signal functions, or comprehensive emergency obstetric care (CEmOC) if they have performed all nine of the signal functions over a defined period. The framework has been enormously influential since the original publication. But much has changed in health systems and the maternal and newborn health landscape since then.

This Delphi study is part of the **Re-Visioning Emergency Obstetric and Newborn Care (EmONC) Project**, an initiative to review, rethink, and revise the EmOC monitoring framework. The Re-Visioning EmONC project is coordinated by the Averting Maternal Death and Disability (AMDD) program at Columbia University Mailman School of Public Health, in collaboration with the London School of Hygiene and Tropical Medicine, UNICEF, UNFPA, and WHO with the aim of creating a revised framework with indicators, tools, and guidance that meet country needs. The entire project is framed and implemented using principles of human-centered design to ensure that the revised framework meets the needs, quality of care standards and real world conditions at the national and sub-national policy level and at the frontlines of health systems in low- and middle-income countries.

The Delphi study on obstetric and newborn signal functions and levels of care is one part of this project. The aim of this Delphi study is to build consensus on the obstetric and newborn signal functions and their organisation across levels of care in low- and middle-income countries. The survey will generate input from clinical and maternal and newborn health experts from different fields and settings worldwide. The project is committed to integrating maternal and newborn care.

The Delphi Survey will include three or more sequential surveys, which will take an estimated 20-30+ minutes each to complete and will be sent out over a period of 3-6 months. Participants who complete**all rounds** of the survey will be offered group authorship on the resulting Delphi study publication.

We are looking for expert assistance with this process. If you would like to be involved, please fill in the following questions to tell us a little about yourself and your expertise for eligibility, to be contacted with the first round survey. All rounds of the survey will be available in English, French and Spanish.  

Thanks!

Dr Sarah Moxon and Dr Sudha Sharma
On behalf of the Re-Visioning EmONC Steering Committee


***The survey is coordinated by the London School of Hygiene & Tropical Medicine and Columbia University as part of the Re-visioning EmONC project, which is funded by a grant from The Bill and Melinda Gates Foundation.***

[1]  https://www.who.int/reproductivehealth/publications/monitoring/9789241547734/en/

2. Consent

Please complete the following: *****

|  | Yes | No |
| --- | --- | --- |
| I consent to provide my name, email address and other information requested in the following questions for the purpose of my possible inclusion in the Delphi study on EmONC signal functions and levels of care. This information will only be used by the survey team to assess my inclusion and to send me the survey. I understand that the information will be permanently deleted from stored files if I do not meet the inclusion criteria, if I inform the survey team that I do not wish to participate, and once the survey is complete. |  |  |

1. Have you ever, or do you currently work in Reproductive, Maternal, Newborn or Child Health (RMNCH)? *****

|  | Yes |
| --- | --- |
|  | No |

4. Information about you

Name (First and Last) *****

|  |
| --- |

Email (please use the same email address in every survey you take part in) *****

|  |
| --- |

2. Are you a:
(select all that apply) *****

|  | 1. Obstetrician-Gynaecologist |
| --- | --- |
|  | 2. Neonatologist |
|  | 3. Pediatrician |
|  | 4. Physician/Medical Doctor |
|  | 5. Nurse |
|  | 6. Neonatal Nurse |
|  | 7. Midwife |
|  | 8. Associate Clinician/Clinical Officer |
|  | 9. Researcher/Academic in Maternal Health |
|  | 10. Researcher/Academic in Newborn Health |
|  | 11. Technical Advisor in Reproductive, Maternal, Newborn or Child Health |
|  | 12. Programme Manager in Reproductive, Maternal, Newborn or Child Health |
|  | 13. Policy-maker/Planner in Reproductive, Maternal, Newborn or Child Health |
|  | 14. Government Official |
|  | 15. Clinical Trainer or Instructor |
|  | 16. Other (please specify):   \|  \| \| --- \| |

3. How many years of experience do you have working in RMNCH? *****

|  | Less than 2 years |
| --- | --- |
|  | 2-5 years |
|  | 6-10 years |
|  | 11-20 years |
|  | More than 20 years |

4. Are you trained as a clinician? *****

|  | Yes |
| --- | --- |
|  | No |

5. Are you currently providing clinical care in Maternal and Newborn Health? *****

|  | Yes |
| --- | --- |
|  | No |

6. In which type of setting(s) are you currently providing clinical care? *****

|  | Community |
| --- | --- |
|  | Health post |
|  | Health centre |
|  | Hospital |
|  | Other (please specify):   \|  \| \| --- \| |

7. In which sector do you provide clinical care? (select all that apply) *****

|  | Public |
| --- | --- |
|  | Private |

8. Which WHO regions of the world do you have significant experience working in Maternal and Newborn Health? (select all that apply) *****

|  | African Region |
| --- | --- |
|  | Americas Region |
|  | Eastern Mediterranean Region |
|  | European Region |
|  | South-East Asian Region |
|  | Western Pacific Region |

9. In which country do you have the most experience working in Maternal and Newborn Health? *****

|  |
| --- |

10. In which country are you currently based? *****

|  |
| --- |

11. Which settings have you worked in? *****

|  | 1. High-income country only |
| --- | --- |
|  | 2. Low and Middle-income country only |
|  | 3. A combination of High and Low and Middle-income countries |

12. Please write the name of the primary institution where you are currently employed/affiliated:

|  |
| --- |

13. Which language would you prefer to take the survey in? *****

|  | English |
| --- | --- |
|  | Spanish |
|  | French |
|  | Other (please specify):   \|  \| \| --- \| |

14. To which gender identity do you most identify? *****

|  | 1. Female |
| --- | --- |
|  | 2. Male |
|  | 3. Transgender female |
|  | 4. Transgender male |
|  | 5. Prefer not to say |
|  | 6. Not listed, option to specify:   \|  \| \| --- \| |

15. Any questions or comments?

|  |
| --- |

**Thank you so much for answering these questions!**

**If you meet the inclusion criteria, a member of the survey team will contact you by email with further information and a link to the first Delphi survey.

If you have any questions at any time, including how your data will be used and stored, please feel free to get in touch with Dr Sarah Moxon, (Sarah.Moxon@lshtm.ac.uk).**
