# Supplementary material for: A Delphi process to build consensus on revised Emergency Obstetric and Newborn Care (EmONC) signal functions and levels of care
Source: PLoS One. 2025 Sep 22;20(9):e0331684. doi: 10.1371/journal.pone.0331684 (PMC12453252; doi:10.1371/journal.pone.0331684)
Supplement: S3 Appendix — (DOCX) [file pone.0331684.s003.docx]

**S3 Appendix: Participant information and R1 survey, including consent form**

**Participant Information Sheet**

**Title of Project: A modified Delphi study on obstetric and newborn care signal functions**

**Introduction**

*The 1997 Guidelines for monitoring the availability and use of obstetric services describe a set of connected indicators to measure and monitor the availability, utilization, and quality of emergency obstetric care in low- and middle-income countries (LMICs). This framework is built around signal functions, which are a shortlist of nine key medical interventions that can "signal" the level of functionality of a health facility. Facilities are subsequently designated as having provided either basic emergency obstetric care (BEmOC) if they can perform seven of the signal functions or comprehensive emergency obstetric care (CEmOC) if they have performed all nine the signal functions over a defined period (typically three months). The framework has been enormously influential over the last 30 years. It is now found in virtually all LMIC health plans for maternal and newborn health (MNH), and often forms the basis for planning for infrastructure, human resources, equipment, supplies, transport, and referral related to care during childbirth. But much has changed in the health systems and MNH landscape in the last 30 years. This modified Delphi study is part of the Re-Visioning EmONC project; an initiative to review, rethink, and revise the EmONC monitoring framework.*

*The Re-Visioning Emergency Obstetric and Newborn Care (EmONC) Project is led by a Steering Committee coordinated by the Averting Maternal Death and Disability (AMDD) program at Columbia University Mailman School of Public Health, and includes the London School of Hygiene and Tropical Medicine, UNICEF, UNFPA, and WHO. The entire project is framed and implemented using principles of human-centered design to ensure that the revised EmONC framework meets the needs and real world conditions at the national and sub-national policy level and at the frontlines of health systems in LMICs at different stages of the obstetric transition.*

*In view of your expertise in maternal and newborn health, we would like to invite you to take part in a Delphi study that we are carrying out as part of the Re-Visioning EmONC project. This information sheet with provide you with more details on the study and what participation will involve.*

**What is the purpose of the study?**

*The purpose of this online modified Delphi study on obstetric and newborn care signal functions is to solicit knowledge and a level of consensus from experts in maternal and newborn health like you in order to review, rethink and revise the existing obstetric care signal functions and levels of care. The project is committed to fully integrating newborn care signal functions as part of the revision.*

**Why have I been asked to take part?**

*You have been invited to join this expert panel because of your clinical and/or health systems expertise in the field of maternal and newborn health. Within the expert panel, we are aiming for experts in a range of roles and from a diverse range of geographies with a combination of ground-level experience, clinical and/or health systems related knowledge in global maternal and newborn health.*

**Do I have to take part?**

*It is up to you to decide to take part or not and we recognise that a Delphi study is a commitment of time. If you agree to join the expert panel and participate in the study, then we will then ask you to sign an online consent form before you can complete the study questions.*

**What is the process for the study and what will I have to do?**

*A Delphi study is a structured process which aims to build consensus by presenting questionnaires to a selected panel of experts in a series of rounds. Rounds are usually repeated until a level of consensus is reached. This modified Delphi study will be carried out through three rounds of online questionnaires. The first questionnaire will be made available on 17th November 2021 and will ask more open questions that allow you to provide free text responses. There will be approximately 8 weeks between subsequent questionnaire rounds.*

*Subsequent questionnaires provide you with feedback on the results from the previous rounds and will ask you to rate signal functions options based on this feedback. We may also provide you with other relevant information or research to help you to respond to the questionnaire. We will allow approximately 8 weeks between rounds aiming to start in November 2021 and aiming to finish in April-May 2022.*

*At the end of the rounds we may hold a final consensus building workshop. Further details on this will be available closer to the time, but we anticipate a commitment of half a day for this workshop.*

**Is my participation confidential?**

*A traditional Delphi study is completely anonymous, however, because this study involves some interaction of participants through a potential workshop at the end of the study complete anonymity of the study is not possible. You may know some of the other participants through professional connections. That is why this study is referred to as a modified Delphi. Whilst participation cannot be fully anonymous, we will anonymise all your responses for the purpose of the analyses and anything you write in free text will be anonymised when collated as feedback. Direct quotes from your responses may be used in the draft study report, but will be anonymised.*

**What are the possible risks and disadvantages?**

*There are very few risks of taking part in this Delphi study other than the commitment of time. Some may find the consensus process challenging and whilst we will do whatever we can to manage diverse opinions, there will always be some compromise to reach consensus. Participants should be aware that whilst they can withdraw from the study at any time and all demographic information will be removed, it may not be possible to remove all contributions that they have made to previous rounds. This is due to the iterative process of the survey and survey tools which may have incorporated some of their inputs into the subsequent rounds of survey tools.*

**What are the possible benefits?**

*Your contribution to the Delphi study will help us to ensure that the revision of the EmONC framework better reflects the current global health landscape and, most importantly, the health system needs of women and newborns. The EmONC framework, widely used in LMIC health planning, has the potential to positively influence health services and subsequent health outcomes for mothers and newborns. Participating in this study helps us to ensure we make informed evidence-based recommendations for the revision of the EmONC framework and allows participants, most of whom will have an interest in the results of the study, to contribute their perspectives to the review, revise and rethink process.*

**What if something goes wrong?**

*If you have a concern about any aspect of this study, you should contact the researchers who will do their best to answer your questions and concerns.*

**Can I change my mind about taking part?**

*You can withdraw at any point, but information collected from previous rounds may still be used as it is not always possible to remove data from previous rounds when it has already been analysed and shared as part of the feedback for each round.*

**What will happen to information collected about me?**

*The information from this study will be analysed and synthesised for this research project. Your personal details, meaning your name and other identifiable information, will be kept in a different safe place to the other study information and will be destroyed within 10 years of the end of the study. At the end of the project, the study data will be archived in the LSHTM data repository* [*https://datacompass.lshtm.ac.uk/information.html*](https://datacompass.lshtm.ac.uk/information.html)*. Your personal information will not be included and there is no way that you can be identified.*

**What will happen to the results of this study?**

*The findings from the Delphi study will be synthesised as part of the Re-Visioning EmONC project and used to recommend a revised framework of signal functions. The study results will be written up as a report and may be written as a publication to be published in a global health journal as part of the study dissemination. You will be given the option for your name to be included in this publication as part of the expert group, but this is optional. If you participate in all rounds of the Delphi, the final draft manuscript will be shared with you and you will be given the option for your name to be included. Otherwise, your personal information will not be included in the study report and there is no way that you can be identified from it.*

**Who is organising and funding this study?**

*This study is funded by LSHTM through a subaward from the Bill and Melinda Gates Foundation to Columbia University Averting Maternal Death and Disability (AMDD) programme.*

**Who has reviewed this study?**

*This study has been reviewed and given favourable opinion by The London School of Hygiene and Tropical Medicine Research Ethics Committee (LSHTM Ethics Ref No. 26292).*

**Further information and contact details**

*Thank you for taking time to read this information sheet. If you think you will take part in the study please read and sign the online consent form before completing the questionnaire.*

*If you have any questions or require any further information, please contact Sarah Moxon sarah.moxon@lshtm.ac.uk*

**Re-Visioning EmONC Delphi Survey Round 1**

1. Introduction

INTRODUCTION

Welcome to Round 1 of the Emergency Obstetric Newborn Care (EmONC) Delphi survey!

**Background**
Twenty-five years ago the novel emergency obstetric care (EmOC) framework provided for the first time [guidelines](https://www.publichealth.columbia.edu/sites/default/files/pdf/unguidelinesen.pdf) for monitoring the availability and use of obstetric services. The set of connected indicators standardised measurement and monitoring of the availability, utilisation, and quality of EmOC in low- and middle-income countries (LMICs). The original EmOC framework, built around signal functions, categorised two levels of care: basic and comprehensive (Figure 1). This common language for policy makers, measurement experts, clinicians and researchers has been enormously influential. The 2009 update (Figure 1) led by Averting Maternal Death and Disability (AMDD) and UN agencies published[Monitoring emergency obstetric care: A Handbook.](https://www.who.int/reproductivehealth/publications/monitoring/9789241547734/en/) Since then, the term "EmONC" has been adopted and the maternal and newborn health (MNH) landscape has continued to progress; this has included an increase in health facility delivery.

**Figure 1. Basic and Comprehensive Emergency Obstetric Care (EmOC) signal functions from 1997 to present**

**Re-Visioning Emergency Obstetric and Newborn Care (EmONC) Project**
The Re-Visioning Emergency Obstetric and Newborn Care (EmONC) Project is led by a Steering Committee coordinated by the Averting Maternal Death and Disability (AMDD) program at Columbia University Mailman School of Public Health, in collaboration with the London School of Hygiene & Tropical Medicine, UNICEF, UNFPA, and WHO. The overall aim is to create a revised framework for obstetric and newborn care with indicators, tools, and guidance that can meet country needs in 2022 and beyond. Separate workstreams in the Re-Visioning EmONC project are looking in detail at: 1) signal functions; 2) levels of care; 3) quality of care, including potential new indicators on readiness and experience of care; and 4) the framework and indicators, as a connected set, informed by country case studies and global experience of their use.

**The Delphi Survey**
A Delphi study is a widely used methodology to build consensus among a panel of experts through a series of interrelated survey rounds. As part of the Re-Visioning EmONC project, this online Delphi survey (LSHTM Ethics Ref No. 26292) seeks to provide input specifically on the A) signal functions and B) levels of care for the framework. Due to your clinical and programme expertise in maternal and newborn health, you are being invited to join this Delphi survey to build consensus on an optimal (but adaptable) service delivery framework that integrates maternal and newborn care. 

This online Delphi survey will include three or more rounds of interrelated surveys, which will be sent out over the next 3-6 months. Each round will take an estimated 20-30+ minutes to complete. Participants who complete all rounds of the survey will be offered collaborative group authorship on the resulting Delphi survey publication.

The first round of this Delphi survey includes open-ended questions that will invite you to share your opinion regarding: 

Section A) Which aspects of maternal and newborn care that the revised signal functions should capture.
Section B) Which criteria should influence how levels of care for maternal and newborn care should be organised.

2. Consent

CONSENT FORM *

|  | Yes | No |
| --- | --- | --- |
| I confirm that I have read and understood the participant information about the modified Delphi study on obstetric and newborn care signal functions and levels of care (sent in the invitation email). I have had the opportunity to consider the information. |  |  |
| I understand that my consent is voluntary and that I am free to withdraw this consent at any time without giving any reason and without my legal rights being affected. |  |  |
| I understand that information submitted in questionnaires will be transcribed and reviewed by researchers in the study and that directly identifiable details will be removed from this transcription for confidentiality. |  |  |
| I understand that information that I submit in the questionnaires for this study will be used by researchers in the study and I give permission for these individuals to have access to my fully anonymised records. |  |  |
| I understand that the study is carried out in a series of rounds and that if I withdraw in a later round of the study it won’t be possible to withdraw information that was provided in previous rounds of the study. |  |  |
| I agree that anonymised direct quotes from my responses may be used in the study report, publications and/or presentations on the results of this study. |  |  |
| I understand that the anonymised data from this study will be stored in a data repository and that access to this data will only be provided at the discretion of the principal investigator by means of a request form. |  |  |
| I consent to provide my email address for the purpose of this study, which will be used to contact me and identify my responses by the study team, and that my email will be deleted from stored files at the end of the study. |  |  |
| I agree to taking part in the above named study. |  |  |

3. Contact Information

1. Have you ever, or do you currently work in Reproductive, Maternal, Newborn or Child Health (RMNCH)? *

|  | Yes |
| --- | --- |
|  | No |

Name (First and Last) *

|  |
| --- |

Email (please use the same email address in every survey you take part in) *

|  |
| --- |

SECTION A: Which aspects of maternal and newborn care should the revised signal functions capture?

SIGNAL FUNCTIONS – A RECAP

The term "signal functions", in this context, means a short list of key interventions intended to "signal" health system capability to deliver a certain level of care.

In 1997 the original list of EmOC signal functions was based on providing emergency obstetric care for complications in childbirth. At the time, most births in LMICs were at home and health facilities were mainly accessed for emergency obstetric care. Now in the sustainable development goal (SDG) era >80% of global births are in health facilities and guidelines and updated standards for improving the quality of care for women and newborns in LMICs have been [published.](https://apps.who.int/iris/bitstream/handle/10665/249155/9789241511216-eng.pdf?sequence=1&isAllowed=y)  

Newborn resuscitation was added as a signal function in 2009 (Figure 1), but it is widely accepted that a greater focus on newborn care is now needed. The Re-Visioning EmONC project aspires to reflect the need for synergy for women and newborns in facility capacity for high-quality, respectful care.

Re-Visioning Signal Functions

The existing EmOC signal functions (1997 and 2009) are based on providing emergency care for obstetric complications. The original signal functions are:

| Inpatient clinical interventions or services (and therefore contain an action verb) e.g. "administer" (which assumes that minimum equipment, drugs and human resources are available) |
| --- |
| Evidence based and/or based on up to date guidelines |
| Indicative of the level of care of a facility |
| Key to averting maternal mortality |

Since the original EmOC signal functions were designed, poor quality of care has been recognised as a major contributor to the slow progress in ending preventable maternal and newborn death and disability, and stillbirths. [Quality of care](https://www.who.int/docs/default-source/mca-documents/advisory-groups/quality-of-care/standards-for-improving-quality-of-maternal-and-newborn-care-in-health-facilities.pdf) has two interrelated dimensions - provision of care and patient experience of care.  Quality of care standards for maternal and newborn care and [small and sick newborns](https://www.who.int/publications/i/item/9789240010765) are published that explicitly define what is required in order to achieve high-quality care.

The Re-Visioning EmONC project is committed to integrating maternal and newborn care in the revised list of signal functions.  

The revised indicator set for the 2022 Re-Visioning EmONC project is expected to include signal functions and separate indicators for quality of care including experience of care as well as new indicators for facility readiness (e.g. infrastructure, referral, human resources).

In this Re-Visioning EmONC project, as well as integrating newborn care more fully, we also have the opportunity to re-visit signal functions and to consider covering aspects of care beyond the management of obstetric emergencies.

2. Type of care

For 2022 onwards, signal functions should cover these types of care (tick all that you think apply). For each one ticked please give your rationale in the comments box below. *

|  | Emergency care for women and newborns |
| --- | --- |
|  | Routine care for women and newborns, including detection and prevention of complications |

Comments:

|  |
| --- |

3. Type of interventions

Signal functions should cover these types of care (tick all that you think apply). For each one ticked please give your rationale in the comments box below.*

|  | Clinical interventions or services (e.g. medical treatments/drugs or procedures) for women and newborns |
| --- | --- |
|  | Non-clinical interventions or services (e.g. include broader dimensions of facility-based care such as organisation of referral) for women and newborns |

Comments:

|  |
| --- |

4. Timing of care

Signal functions should cover these areas of care (tick all that you think apply). For each one ticked please give your rationale in the comments box below. *

|  | Intrapartum care (for both women and newborns) |
| --- | --- |
|  | Care during pregnancy |
|  | Postnatal care (for both women and newborns) |
|  | Care for small and sick newborns |
|  | Post abortion care |
|  | Other (please specify):   \|  \| \| --- \| |

Comments:

|  |
| --- |

5. If we maintain current assumptions based on signal functions as interventions/services to treat complications:

| a) Which priority complications for women should be included? (Include as many as you wish) | \|  \| \| --- \| |
| --- | --- | --- |
| b) Which priority complications for newborns should be included? (Include as many as you wish) | \|  \| \| --- \| |

SECTION B. Which criteria should influence how levels of maternal and newborn care should be organised

The original EmOC signal function framework (1997 and 2009) was based on two levels of care, basic and comprehensive. These levels reflected health system capability to deliver care determined by signal functions of interventions to treat complications: eight obstetric and one neonatal. These two levels of facility care also broadly correspond to competencies for midwives (basic EmOC) or physicians (comprehensive EmOC). 

Levels of care can be organised around interventions/signal functions or other criteria. The Re-Visioning EmONC project is committed to exploring an optimal configuration of levels of care both within and between health facilities for both women and newborns.

6. If you think about defining levels of care for women and newborns, which important health system criteria should be considered?

Are interventions/signal functions sufficient to differentiate levels of care or which other criteria should be considered?

|  |
| --- |

7. Linkage between maternal and newborn levels of care

The 2009 signal function framework has maternal and newborn signal functions grouped together for levels of care.

Should the revised 2022 framework group maternal and newborn signal functions together or separately for levels of care? Please explain your rationale in the comments box. *

|  | Together |
| --- | --- |
|  | Separately |
|  | No preference |

Comments:

|  |
| --- |

8. Current levels of care for women and newborns in national health systems
Think about the levels in your national health system (or a system you are very familiar with). Some countries have 2 or 3 levels, others have 6 or more.

Please describe how many levels of care there are in the setting you work in or know best and fill in one criterion of care for women and one for newborns that is used to designate these levels. Please name the levels beginning with level 1 as the lowest health system level, then level 2 as the next level up until all levels are numbered.

Please add any comments in the comments box below the table.

|  | Name of level | Women | Newborn |
| --- | --- | --- | --- |
|  | \|  \| \| --- \| | \|  \| \| --- \| | \|  \| \| --- \| |
|  | \|  \| \| --- \| | \|  \| \| --- \| | \|  \| \| --- \| |
|  | \|  \| \| --- \| | \|  \| \| --- \| | \|  \| \| --- \| |

Comments:

|  |
| --- |

9. Optimal configuration of levels of care
Newborn care levels have recently been envisaged with 3 levels plus a transitional level between levels 2 and 3 (Figure 2). Transition means delivering more care than a lower level, but not all the care required to classify as a higher level of care.

Figure 2. Newborn care interventions by health system levels


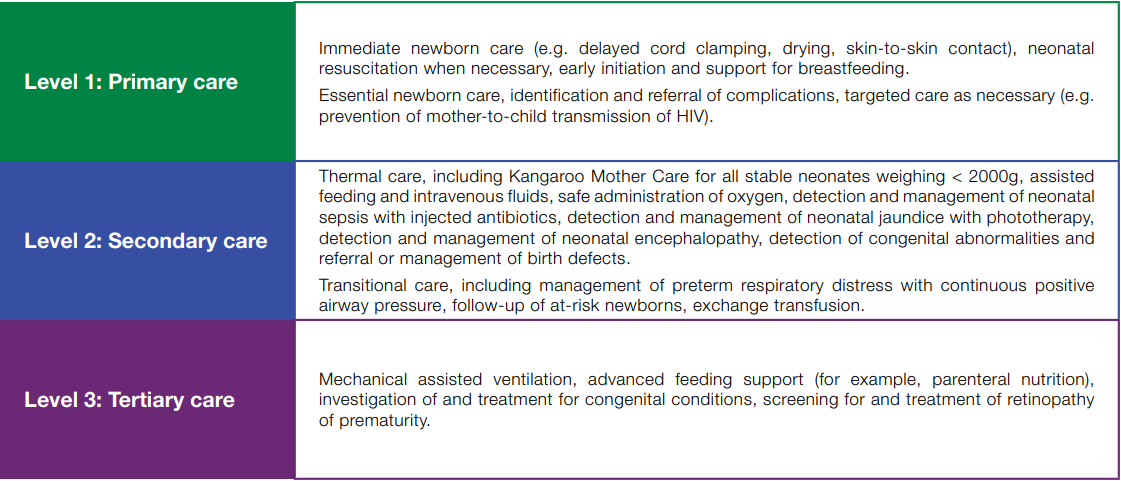


Source: World Health Organization. Survive and Thrive Transforming care for every small and sick newborn. 2019. https://apps.who.int/iris/bitstream/handle/10665/326495/9789241515887-eng.pdf(accessed 25 Jan 2021).

What is the optimal number of levels of care for women and newborns with complications? Please give your rationale in the comments box below. *

|  | 2 levels |
| --- | --- |
|  | 3 levels |
|  | 3 levels plus one transition level between level 2 and level 3 (total 4 levels) |
|  | 3 levels plus one transition level between level 2 and level 3 and one transition between level 1 and 2 (total 5 levels) |
|  | More levels- please specify how many levels below |

Comments:

|  |
| --- |

8. Information about you

To ensure that our survey includes a diverse panel of experts, please complete the section below to tell us a bit about yourself.

10. Are you a:
(select all that apply) *

|  | 1. Obstetrician-Gynaecologist |
| --- | --- |
|  | 2. Neonatologist |
|  | 3. Paediatrician |
|  | 4. Physician/Medical Doctor |
|  | 5. Nurse |
|  | 6. Neonatal Nurse |
|  | 7. Midwife |
|  | 8. Associate Clinician/Clinical Officer |
|  | 9. Researcher/Academic in Maternal Health |
|  | 10. Researcher/Academic in Newborn Health |
|  | 11. Technical Advisor in Reproductive, Maternal, Newborn or Child Health |
|  | 12. Programme Manager in Reproductive, Maternal, Newborn or Child Health |
|  | 13. Policy-maker/Planner in Reproductive, Maternal, Newborn or Child Health |
|  | 14. Government Official |
|  | 15. Clinical Trainer or Instructor |
|  | 16. Other (please specify):   \|  \| \| --- \| |

11. How many years of experience do you have working in RMNCH? *

|  | Less than 2 years |
| --- | --- |
|  | 2-5 years |
|  | 6-10 years |
|  | 11-20 years |
|  | More than 20 years |

12. Are you trained as a clinician? *

|  | Yes |
| --- | --- |
|  | No |

13. Are you currently providing clinical care in Maternal and Newborn Health? *

|  | Yes |
| --- | --- |
|  | No |

14. In which type of setting(s) are you currently providing clinical care? *

|  | Community |
| --- | --- |
|  | Health post |
|  | Health centre |
|  | Hospital |
|  | Other (please specify):   \|  \| \| --- \| |

15. In which sector do you provide clinical care? (select all that apply) *

|  | Public |
| --- | --- |
|  | Private |

16. Which WHO regions of the world do you have significant experience working in Maternal and Newborn Health? (select all that apply) *

|  | African Region |
| --- | --- |
|  | Americas Region |
|  | Eastern Mediterranean Region |
|  | European Region |
|  | South-East Asian Region |
|  | Western Pacific Region |

17. In which country do you have the most experience working in Maternal and Newborn Health? *

18. In which country are you currently based? *

19. Which settings have you worked in? *

|  | 1. High-income country only |
| --- | --- |
|  | 2. Low and Middle-income country only |
|  | 3. A combination of High and Low and Middle-income countries |

20. Please write the name of the primary institution where you are currently employed/affiliated:

|  |
| --- |

21. To which gender identity do you most identify? *

|  | 1. Female |
| --- | --- |
|  | 2. Male |
|  | 3. Transgender female |
|  | 4. Transgender male |
|  | 5. Prefer not to say |
|  | 6. Not listed, option to specify:   \|  \| \| --- \| |

Thank you so much for completing the Round 1 Survey!

We will email you a link to the Round 2 Survey in the next 1-2 months. If you have any questions, please contact: Sarah.Moxon@lshtm.ac.uk. 

Best regards,
Dr Sarah Moxon and Dr Sudha Sharma
On behalf of the EmONC Steering Committee
