# Supplementary material for: A Delphi process to build consensus on revised Emergency Obstetric and Newborn Care (EmONC) signal functions and levels of care
Source: PLoS One. 2025 Sep 22;20(9):e0331684. doi: 10.1371/journal.pone.0331684 (PMC12453252; doi:10.1371/journal.pone.0331684)
Supplement: S4 Appendix — (DOCX) [file pone.0331684.s004.docx]

**S4 Appendix: R2 survey**

Delphi study on obstetric and newborn signal functions and levels of care: ROUND 2

1. Introduction

**Delphi study on obstetric and newborn care signal functions for the Re-Visioning Emergency Obstetric and Newborn care (EmONC) Project**

Thank you for your participation in round one of the Delphi study on obstetric and newborn care signal functions and levels of care for the Re-Visioning EmONC project. In total, 212 experts participated, sharing many insightful responses and comments.

**Welcome to Round Two!**

**Background**

Twenty-five years ago, the novel emergency obstetric care (EmOC) framework provided for the first time [**guidelines**](https://www.publichealth.columbia.edu/sites/default/files/pdf/unguidelinesen.pdf) for monitoring the availability and use of obstetric services. The set of connected indicators standardised measurement and monitoring of the availability, utilisation, and quality of EmOC in low- and middle-income countries (LMICs). The original EmOC framework, built around ***signal functions***, categorised two ***levels of care***: basic and comprehensive (Figure 1). This common language for policy makers, program planners, measurement experts, clinicians and researchers has been enormously influential. Since then, the term "EmONC" has been adopted and the maternal and newborn health (MNH) landscape has continued to progress; this has included an increase in health facility delivery.

**Figure 1. Basic and Comprehensive Emergency Obstetric Care (EmOC) signal functions from 1997 to present**


**The Re-Visioning Emergency Obstetric and Newborn Care (EmONC) Project**

The Re-Visioning Emergency Obstetric and Newborn Care (EmONC) Project is led by a Steering Committee coordinated by the Averting Maternal Death and Disability (AMDD) program at Columbia University Mailman School of Public Health, in collaboration with UNICEF, UNFPA, WHO and the London School of Hygiene & Tropical Medicine. The overall aim is to create a revised framework for obstetric and newborn care with indicators, tools, and guidance that can meet country needs in 2022 and beyond.

**The Delphi study**

A Delphi study is a widely used methodology to build consensus among a panel of experts through a series of interrelated survey rounds; this is round two of what we expect to be a total of three rounds. As part of the Re-Visioning EmONC project, the overall aim of this Delphi study (LSHTM Ethics Ref No. 26292) is to build consensus on maternal and newborn signal functions and levels of care for a revised EmONC framework.

**Round Two of the Delphi Survey**

Round two of this Delphi study is focused on **prioritising obstetric and newborn emergency signal functions** for the revised EmONC framework. We will provide a clear definition of signal functions and their desired characteristics and ask you to select priority emergency signal functions for the revised framework based on these.

The content presented for this round of the Delphi is grounded in the results from the first round of this Delphi study, as well as background work, which was refined during a Re-Visioning EmONC technical workshop in May 2022.  

***What are emergency signal functions?***

Emergency signal functions are: **a parsimonious list of clinical tracer interventions, representing key processes of care, to treat the main complications of childbirth that would otherwise result in maternal or newborn death and disability, or stillbirth.** They are a simple measure of ***whether the facility performed the function.*** Signal functions are usually captured through surveys and/or routine data collection processes at the facility level.

***What else will be in the revised EmONC framework?***

Emergency signal functions will be used as part of the revised EmONC framework to monitor the availability of emergency obstetric services and small and sick newborn care. The framework will also include additional input indicators such as infrastructure, drugs, and equipment, as well as output and outcome indicators that assess access and quality, including experience of care. Routine care signal functions such as essential newborn care and active management of third stage of labour (AMTSL) will also be included as a separate domain of the revised framework, but are not included in this round, which focuses on emergency signal functions. **All these additional indicators are not part of the signal functions being addressed in this Delphi round two, which focuses only on emergency care signal functions.**

***What are the key characteristics of emergency obstetric and newborn signal functions?***

An ideal obstetric and newborn care emergency signal function for the revised framework should be:

|  **CRITICAL:** The signal function should represent a clinical intervention or service performed by health workers to manage one or more complication(s) that would otherwise result in major cause/s of maternal or newborn death and disability, or stillbirth.   **a TRACER:** Performance of the intervention or service ideally indicates that multiple other components of treatment or aspects of care are also present (e.g., performance of surgery generally indicates the availability of anaesthesia).   **FREQUENT:**The intervention or service should be performed often enough to reflect ongoing performance or functionality of the facility.   **SIMPLE:** Not necessarily simple to perform, but the intervention or service should be clearly and operationally defined and feasible to measure reliably across contexts.* |
| --- |

*For new signal functions (especially some newborn signal functions) it might be several years before they can be measured reliably in many contexts, therefore, the proposed signal function should be a clearly defined intervention such that in future it can be measured reliably across contexts.

2. Your information

Name (First and Last, as you entered it in the round one survey): *****

|  |
| --- |

Email (please use the same email address that you used in the round one survey): *****

|  |
| --- |

In the past, for which purpose(s) have you used the EmONC framework? Please describe in the comments box below. *****

|  |
| --- |

3. Please select your sections

The first section presents the proposed emergency obstetric care signal functions, the second section presents the small and sick newborn signal functions. Click to select the section(s) you would like to complete. *****

|  | Obstetric |
| --- | --- |
|  | Newborn |
|  | Obstetric and newborn |

**Emergency obstetric care signal functions**
***As you review these emergency signal functions, consider the following context:***

Imagine you are working in a managerial or planning role at a district hospital in a low- or middle-income setting that you know well. The facility has a delivery caseload of at least 1000 births per year. There is a labour and delivery ward, an obstetric surgery operating room, and a neonatal unit. Think about the type of supplies, equipment, and staffing capacity typically available as well as some of the regular challenges facing the facility in this context.

Listed below are ***emergency obstetric signal functions.*** Choose the nine most important emergency signal functions for the low- and middle income facility context you imagined. While prioritising, consider the key characteristics of a signal function for the revised framework as defined below: 

**CRITICAL:**The signal function should represent a clinical intervention or service performed by health workers to manage one or more complication(s) that would otherwise result in major cause/s of maternal or newborn death and disability, or stillbirth.
**a TRACER:** Performance of the intervention or service ideally indicates that multiple other components of treatment or aspects of care are also present (e.g., performance of surgery generally indicates the availability of anaesthesia).
**FREQUENT:** The intervention or service should be performed often enough to reflect ongoing performance or functionality of the facility.
**SIMPLE:** Not necessarily simple to perform, but the intervention or service should be clearly and operationally defined and feasible to measure reliably across contexts.*

*For new signal functions (especially some newborn signal functions) it might be several years before they can be measured reliably in many contexts, therefore, the proposed signal function should be a clearly defined intervention such that in future it can be measured reliably across contexts.

1. Drag and drop (or number) the top nine signal functions that you think should be prioritised for the revised EmONC framework based on the information above. The numbers 1-9 will be used to count your top nine signal functions, not to rank them in order of importance.

The document attached to your email invitation contains a table with additional information for each proposed signal function. You can also view the document using this QR code:
Drag and drop (or number) your top nine below: *****

| Administer parenteral antibiotics (e.g. ampicillin, gentamicin) (maternal) | \|  \| \| --- \| |
| --- | --- | --- |
| Administer appropriate medications to treat post-partum haemorrhage (PPH) (appropriate medications in the algorithm for PPH e.g. oxytocin or ergometrine (or combination of oxytocin and ergometrine), or oral misoprostol, or prostaglandin or heat stable carbetocin or tranexamic acid). | \|  \| \| --- \| |
| Administer magnesium sulfate for pre-eclampsia or eclampsia | \|  \| \| --- \| |
| Administer maternal antihypertensives (e.g. alpha methyldopa, hydralazine, labetalol, nifedipine) | \|  \| \| --- \| |
| Provide intravenous (IV) infusion/IV fluid replacement therapy (e.g. maternal resuscitation) | \|  \| \| --- \| |
| Perform manual uterine exploration and removal of placenta | \|  \| \| --- \| |
| Perform removal of retained products of conception (e.g. manual vacuum aspiration), dilation and curettage, medical management) | \|  \| \| --- \| |
| Perform assisted vaginal birth (e.g. vacuum extractor, forceps) | \|  \| \| --- \| |
| Perform blood transfusion | \|  \| \| --- \| |
| Perform caesarean section | \|  \| \| --- \| |
| Perform mechanical ventilation | \|  \| \| --- \| |
| Provide high dependency/intensive-care | \|  \| \| --- \| |
| Provision of continued emergency clinical obstetric and newborn care during interfacility transfer | \|  \| \| --- \| |

Is there an additional emergency obstetric signal function that you think is essential to add? If so, please list below.

|  |
| --- |

Do you have any comments/suggestions on the wording of any of the signal functions?

|  |
| --- |

**Small and Sick Newborn Care Signal Functions**
***As you review these small and sick newborn care signal functions, consider the following context:***

Imagine you are working in a managerial or planning role at a district hospital in a low- or middle-income setting that you know well. The facility has a delivery caseload of at least 1000 births per year. There is a labour and delivery ward, an obstetric surgery operating room, and a neonatal unit. Think about the type of supplies, equipment, and staffing capacity typically available as well as some of the regular challenges facing the facility in this context.

Listed below are ***small and sick newborn signal functions.*** Choose the nine most important signal functions for the LMIC facility context you imagined. While prioritising, consider the key characteristics of a signal function for the revised framework as defined below: 

**CRITICAL:**The signal function should represent a clinical intervention or service performed by health workers to manage one or more complication(s) that would otherwise result in major cause/s of maternal or newborn death and disability, or stillbirth.
**a TRACER:** Performance of the intervention or service ideally indicates that multiple other components of treatment or aspects of care are also present (e.g., performance of surgery generally indicates the availability of anaesthesia). 
**FREQUENT:** The intervention or service should be performed often enough to reflect ongoing performance or functionality of the facility.
**SIMPLE:** Not necessarily simple to perform, but the intervention or service should be clearly and operationally defined and feasible to measure reliably across contexts.*

*For new signal functions (especially some newborn signal functions) it might be several years before they can be measured reliably in many contexts, therefore, the proposed signal function should be a clearly defined intervention such that in future it can be measured reliably across contexts.

2. Drag and drop (or number) the top nine signal functions that you think should be prioritised for the revised EmONC framework based on the information above. The numbers 1-9 will be used to count your top nine signal functions, not to rank them in order of importance.
The document attached to your email invitation contains a table with additional information for each proposed signal function. You can also view the document using this QR code:
Drag and drop (or number) your top nine below: *****

| Administer antenatal corticosteroids (ACS) (e.g. dexamethasone, betamethasone) to women at risk of preterm birth | \|  \| \| --- \| |
| --- | --- | --- |
| Administer magnesium sulfate to women at risk of preterm birth | \|  \| \| --- \| |
| Perform neonatal resuscitation with bag and mask | \|  \| \| --- \| |
| Initiate kangaroo mother care (for LBW/preterm newborns) | \|  \| \| --- \| |
| Administer oxygen therapy with pulse oximetry | \|  \| \| --- \| |
| Administer parenteral antibiotics (e.g. gentamicin, ampicillin) (newborn) | \|  \| \| --- \| |
| Perform assisted feeding with expressed breastmilk (e.g. cup and/or nasogastric feeding) | \|  \| \| --- \| |
| Administer phototherapy for hyperbilirubinemia (jaundice) | \|  \| \| --- \| |
| Administer parenteral anticonvulsants for seizures (newborn) (e.g. phenobarbitone) | \|  \| \| --- \| |
| Perform blood transfusion (newborn) | \|  \| \| --- \| |
| Administer CPAP (newborn) | \|  \| \| --- \| |
| Perform services for retinopathy of prematurity | \|  \| \| --- \| |
| Administer intravenous (IV) fluids (newborn) | \|  \| \| --- \| |
| Provide thermal care (e.g. radiant warmer, incubator, heated cot) for preterm/LBW newborns | \|  \| \| --- \| |
| Administer methylxanthines (e.g. caffeine) for preterm newborns for prevention and treatment of apnoea | \|  \| \| --- \| |
| Provision of continued emergency clinical obstetric and newborn care during interfacility transfer | \|  \| \| --- \| |

Is there an additional small and sick newborn signal function that you think is essential to add? If so, please list below.

|  |
| --- |

Do you have any comments/suggestions on the wording of any of the signal functions?

|  |
| --- |

**Emergency obstetric care signal functions**

***As you review these emergency signal functions, consider the following context:***

Imagine you are working in a managerial or planning role at a district hospital in a low- or middle-income setting that you know well. The facility has a delivery caseload of at least 1000 births per year. There is a labour and delivery ward, an obstetric surgery operating room, and a neonatal unit. Think about the type of supplies, equipment, and staffing capacity typically available as well as some of the regular challenges facing the facility in this context.

Listed below are ***emergency obstetric signal functions.*** Choose the nine most important emergency signal functions for the low- or middle-income facility context you imagined. While prioritising, consider the key characteristics of a signal function for the revised framework as defined below: 

**CRITICAL:**The signal function should represent a clinical intervention or service performed by health workers to manage one or more complication(s) that would otherwise result in major cause/s of maternal or newborn death and disability, or stillbirth.
**a TRACER:** Performance of the intervention or service ideally indicates that multiple other components of treatment or aspects of care are also present (e.g., performance of surgery generally indicates the availability of anaesthesia).
**FREQUENT:** The intervention or service should be performed often enough to reflect ongoing performance or functionality of the facility.
**SIMPLE:** Not necessarily simple to perform, but the intervention or service should be clearly and operationally defined and feasible to measure reliably across contexts.*

*For new signal functions (especially some newborn signal functions) it might be several years before they can be measured reliably in many contexts, therefore, the proposed signal function should be a clearly defined intervention such that in future it can be measured reliably across contexts.

3. Drag and drop (or number) the top nine signal functions that you think should be prioritised for the revised EmONC framework based on the information above. The numbers 1-9 will be used to count your top nine signal functions, not to rank them in order of importance.

The document attached to your email invitation contains a table with additional information for each proposed signal function. You can also view the document using this QR code:
Drag and drop (or number) your top nine below: *****

| Administer parenteral antibiotics (e.g. ampicillin, gentamicin) (maternal) | \|  \| \| --- \| |
| --- | --- | --- |
| Administer appropriate medications to treat post-partum haemorrhage (PPH) (appropriate medications in the algorithm for PPH e.g. oxytocin or ergometrine (or combination of oxytocin and ergometrine), or oral misoprostol, or prostaglandin or heat stable carbetocin or tranexamic acid). | \|  \| \| --- \| |
| Administer magnesium sulfate for pre-eclampsia or eclampsia | \|  \| \| --- \| |
| Administer maternal antihypertensives (e.g. alpha methyldopa, hydralazine, labetalol, nifedipine) | \|  \| \| --- \| |
| Provide intravenous (IV) infusion/IV fluid replacement therapy (e.g. maternal resuscitation) | \|  \| \| --- \| |
| Perform manual uterine exploration and removal of placenta | \|  \| \| --- \| |
| Perform removal of retained products of conception (e.g. manual vacuum aspiration, dilation and curettage, medical management) | \|  \| \| --- \| |
| Perform assisted vaginal birth (e.g. vacuum extractor, forceps) | \|  \| \| --- \| |
| Perform blood transfusion | \|  \| \| --- \| |
| Perform caesarean section | \|  \| \| --- \| |
| Perform mechanical ventilation | \|  \| \| --- \| |
| Provide high dependency/intensive-care | \|  \| \| --- \| |
| Provision of continued emergency clinical obstetric and newborn care during interfacility transfer | \|  \| \| --- \| |

Is there an additional emergency obstetric signal function that you think is essential to add? If so, please list below.

|  |
| --- |

Do you have any comments/suggestions on the wording of any of the signal functions?

|  |
| --- |

**Small and Sick Newborn Care Signal Functions**

***As you review these small and sick newborn signal functions, consider the following context:***

Imagine you are working in a managerial or planning role at a district hospital in a low- or middle-income setting that you know well. The facility has a delivery caseload of at least 1000 births per year. There is a labour and delivery ward, an obstetric surgery operating room, and a neonatal unit. Think about the type of supplies, equipment, and staffing capacity typically available as well as some of the regular challenges facing the facility in this context.

Listed below are ***small and sick newborn signal functions.*** Choose the nine most important emergency signal functions for the low- or middle-income facility context you imagined. While prioritising, consider the key characteristics of a signal function for the revised framework as defined below: 

**CRITICAL:**The signal function should represent a clinical intervention or service performed by health workers to manage one or more complication(s) that would otherwise result in major cause/s of maternal or newborn death and disability, or stillbirth.
**a TRACER:** Performance of the intervention or service ideally indicates that multiple other components of treatment or aspects of care are also present (e.g., performance of surgery generally indicates the availability of anaesthesia). 
**FREQUENT:** The intervention or service should be performed often enough to reflect ongoing performance or functionality of the facility.
**SIMPLE:** Not necessarily simple to perform, but the intervention or service should be clearly and operationally defined and feasible to measure reliably across contexts.*

*For new signal functions (especially some newborn signal functions) it might be several years before they can be measured reliably in many contexts, therefore, the proposed signal function should be a clearly defined intervention such that in future it can be measured reliably across contexts.

4. Drag and drop (or number) the top nine signal functions that you think should be prioritised for the revised EmONC framework based on the information above. The numbers 1-9 will be used to count your top nine signal functions, not to rank them in order of importance.

The document attached to your email invitation contains a table with additional information for each proposed signal function. You can also view the document using this QR code:
Drag and drop (or number) your top nine below: *****

| Administer antenatal corticosteroids (ACS) (e.g. dexamethasone, betamethasone) to women at risk of preterm birth | \|  \| \| --- \| |
| --- | --- | --- |
| Administer magnesium sulfate to women at risk of preterm birth | \|  \| \| --- \| |
| Perform neonatal resuscitation with bag and mask | \|  \| \| --- \| |
| Initiate kangaroo mother care (for LBW/preterm newborns) | \|  \| \| --- \| |
| Administer oxygen therapy with pulse oximetry | \|  \| \| --- \| |
| Administer parenteral antibiotics (e.g. gentamicin, ampicillin) (newborn) | \|  \| \| --- \| |
| Perform assisted feeding with expressed breastmilk (e.g. cup and/or nasogastric feeding) | \|  \| \| --- \| |
| Administer phototherapy for hyperbilirubinemia (jaundice) | \|  \| \| --- \| |
| Administer parenteral anticonvulsants for seizures (newborn) (e.g. phenobarbitone) | \|  \| \| --- \| |
| Perform blood transfusion (newborn) | \|  \| \| --- \| |
| Administer CPAP (newborn) | \|  \| \| --- \| |
| Perform services for retinopathy of prematurity | \|  \| \| --- \| |
| Administer intravenous (IV) fluids (newborn) | \|  \| \| --- \| |
| Provide thermal care (e.g. radiant warmer, incubator, heated cot) for preterm/LBW newborns | \|  \| \| --- \| |
| Administer methylxanthines (e.g. caffeine) for preterm newborns for prevention and treatment of apnoea | \|  \| \| --- \| |
| Provision of continued emergency clinical obstetric and newborn care during interfacility transfer | \|  \| \| --- \| |

Is there an additional small and sick newborn signal function that you think is essential to add? If so, please list below.

|  |
| --- |

Do you have any comments/suggestions on the wording of any of the signal functions?

|  |
| --- |

**Thank you so much for completing the round two survey!**

Participants who complete all rounds of the survey will be offered collaborative group authorship on the resulting Delphi survey publication.

Once analysis of round two is complete, we will contact you with a link to the round three survey. If you have any questions, please contact: Sarah.Moxon@lshtm.ac.uk. 

Best regards,
Dr Sarah Moxon, Dr Sudha Sharma and Dr Jalemba Aluvaala
On behalf of the Re-Visioning EmONC Project Steering Committee
