# Supplementary material for: A Delphi process to build consensus on revised Emergency Obstetric and Newborn Care (EmONC) signal functions and levels of care
Source: PLoS One. 2025 Sep 22;20(9):e0331684. doi: 10.1371/journal.pone.0331684 (PMC12453252; doi:10.1371/journal.pone.0331684)
Supplement: S5 Appendix — (DOCX) [file pone.0331684.s005.docx]

**S5 Appendix: R3 survey**

**Delphi study on obstetric and newborn signal functions and levels of care: ROUND 3**

1. Introduction

**Delphi study on obstetric and newborn care signal functions for the Re-Visioning Emergency Obstetric and Newborn care (EmONC) Project**

Thank you for your participation in rounds one and two of the Delphi study on obstetric and newborn care signal functions and levels of care for the Re-Visioning EmONC project. In total, 131 experts participated in round two, which was focused on prioritising emergency obstetric and newborn care signal functions for the revised EmONC framework.

Welcome to Round Three!

**Background**

Twenty-five years ago, the novel emergency obstetric care (EmOC) framework provided for the first time [guidelines](https://www.publichealth.columbia.edu/sites/default/files/pdf/unguidelinesen.pdf) for monitoring the availability and use of obstetric services. The set of connected indicators standardised measurement and monitoring of the availability, utilisation, and quality of EmOC in low- and middle-income countries (LMICs). The original EmOC framework, built around signal functions, categorised two levels of care: basic and comprehensive (Figure 1). This common language for policy makers, program planners, measurement experts, clinicians and researchers has been enormously influential. With increased recognition of the importance of newborns in global health, the term "EmONC" has been adopted for programme implementation and the maternal and newborn health (MNH) landscape has continued to progress; this has included an increase in health facility delivery.  

**Figure 1. Basic and Comprehensive Emergency Obstetric Care (EmOC) signal functions from 1997 to present**

**The Re-Visioning Emergency Obstetric and Newborn Care (EmONC) Project**

The Re-Visioning Emergency Obstetric and Newborn Care (EmONC) Project is led by a Steering Committee coordinated by the Averting Maternal Death and Disability (AMDD) program at Columbia University Mailman School of Public Health, in collaboration with UNICEF, UNFPA, WHO and the London School of Hygiene & Tropical Medicine (LSHTM). The overall aim is to create a revised framework for obstetric and newborn care with indicators, tools, and guidance that can meet country needs in 2023 and beyond.

**The Delphi study on obstetric and newborn signal functions and levels of care**

A Delphi study is a widely used method to build consensus among experts through a series of interrelated survey rounds. The first round of the Delphi study on obstetric and newborn signal functions and levels of care was carried out in November-December 2021, the second round in August-September 2022, and this is the third and final of three rounds. As part of the Re-Visioning EmONC project, the overall aim of this Delphi study is to build consensus on obstetric and newborn signal functions and levels of care. The study has ethical approval from LSHTM Ethics Ref No. 26292.

**Round three of the Delphi study**

The objective of round three of this Delphi study is to build consensus on a configuration of EmONC signal functions linked to specific levels of care. You will be invited to answer questions and provide feedback on a prototype (a preliminary model) of the signal functions and the recommended levels of care at which these are provided. We anticipate that this will take 20 minutes to complete.

The prototype presented in this round is grounded in results from the previous two rounds of the Delphi study and from a Re-Visioning EmONC Project technical workshop held in September 2022.

**What are emergency signal functions?**

Emergency signal functions are: a parsimonious list of clinical tracer interventions, representing key processes of care, to treat the main complications of childbirth that would otherwise result in maternal or newborn death and disability, or stillbirth. Signal functions are a simple measure of whether the facility performed the function in a specified time period; they are usually captured through surveys and/or routine health information systems at the facility level.

**What are the EmONC signal functions used for?**

EmOC signal functions have been primarily used for planning purposes, to ascertain the availability of basic and comprehensive level EmOC facilities across a population. The Re-Visioning EmONC Project aims to integrate emergency newborn care signal functions to be used for the same purpose. Emergency signal functions are tracers – they do not capture all interventions performed or act as indicators of quality of care. In addition to their use for planning, signal functions have also been used for research, advocacy, accountability, and for training programmes.

**What else will be in the revised EmONC framework?**

In addition to signal functions, the revised EmONC framework will also include a set of updated indicators including input (e.g. infrastructure, drugs, and equipment), output and outcome indicators to assess access and quality, including experience of care. Routine care for women and newborns will also be included as a separate part of the revised framework, but is not included in this Delphi study, which focuses on emergency signal functions and levels of care.

2. Your information

Name (First and Last, as you entered it in the round one survey): *

|  |
| --- |

Email (please use the same email address that you used in the round one survey): *

|  |
| --- |

3. Your opinion on signal functions and levels of care

Emergency obstetric and newborn care (EmONC) signal functions by three levels of care: A prototype


*These signal functions are required at comprehensive and intensive EmONC levels, but they can (and often will) be part of service delivery at first line EmONC facilities.

The document attached to your email invitation contains a table with additional information for each proposed signal function.

**Section A. Levels of care**

This section is about the proposed organisation of signal functions into three levels of care. In round one of this Delphi study, 85% of participants indicated a configuration of signal functions built around three levels of care would be optimal for the revised EmONC framework.

1. What is your opinion on the proposal to use three levels of care as shown in the prototype above?
(This question is not about which signal functions are in each level in the prototype; the signal functions themselves will be discussed in the next section. We simply wish to know your opinion on using three levels of care, as compared to fewer or more levels.) *

|  | Strongly disagree |
| --- | --- |
|  | Disagree |
|  | Agree |
|  | Strongly agree |

If you disagree or strongly disagree please state your reasons in the text box below:

|  |
| --- |

2. In the prototype, we have proposed naming the three levels (first line, comprehensive, intensive).

What is your opinion on naming the lowest level of care "first line"? *

|  | Strongly disagree |
| --- | --- |
|  | Disagree |
|  | Agree |
|  | Strongly agree |

If you disagree or strongly disagree please state your reasons in the text box below:

|  |
| --- |

3. What is your opinion on naming the middle level of care "comprehensive"? *

|  | Strongly disagree |
| --- | --- |
|  | Disagree |
|  | Agree |
|  | Strongly agree |

If you disagree or strongly disagree please state your reasons in the text box below:

|  |
| --- |

4. What is your opinion on naming the highest level of care "intensive"? *

|  | Strongly disagree |
| --- | --- |
|  | Disagree |
|  | Agree |
|  | Strongly agree |

If you disagree or strongly disagree please state your reasons in the text box below:

|  |
| --- |

5. Do you have any other general comments about the naming of the three levels?

|  |
| --- |

4. Select which sections to complete

**Section B. Signal functions**
This section is about the proposed signal functions for the three levels of care. Please choose whether you would like to complete the sections on obstetric signal functions, newborn signal functions or obstetric and newborn signal functions for levels of care. *

|  | Obstetric signal functions |
| --- | --- |
|  | Newborn signal functions |
|  | Obstetric and newborn signal functions |

5. Obstetric

Obstetric care signal functions

An ideal EmONC signal function for the revised EmONC framework should meet these criteria: 

CRITICAL: The signal function should represent a clinical intervention or service performed by health workers to manage one or more complication(s) that would otherwise result in major cause/s of maternal or newborn death and disability, or stillbirth.
a TRACER: Performance of the intervention or service ideally indicates that multiple other components of treatment or aspects of care are also present (e.g., performance of surgery generally indicates the availability of anaesthesia).
FREQUENT: The intervention or service should be performed often enough to reflect ongoing performance or functionality of the facility.
SIMPLE: Not necessarily simple to perform, but the intervention or service should be clearly and operationally defined and feasible to measure reliably across contexts.

Emergency obstetric and newborn care (EmONC) signal functions by three levels of care: A prototype

*These signal functions are required at comprehensive and intensive EmONC levels, but they can (and often will) be part of service delivery at first line EmONC facilities.

6. Based on the above criteria, the following obstetric signal functions should be included in the revised EmONC framework: *

|  | Agree | Disagree |
| --- | --- | --- |
| Administer parenteral antibiotics (maternal) |  |  |
| Administer medications to treat post-partum-haemorrhage (PPH) |  |  |
| Administer magnesium sulfate for severe pre-eclampsia or eclampsia |  |  |
| Provide IV fluid replacement therapy (e.g. for shock or sepsis) |  |  |
| Perform manual removal of retained placenta and uterine exploration |  |  |
| Perform removal of retained products of conception (POC) for abortion or post-abortion care (e.g., vacuum aspiration, dilation and evacuation, medical management) |  |  |
| Perform assisted vaginal birth (e.g. vacuum extractor, ventouse, forceps) |  |  |
| Perform blood transfusion |  |  |
| Perform caesarean section |  |  |
| Provide intensive level organ support |  |  |
| Provide continued clinical care during interfacility transfer |  |  |

Comments:

|  |
| --- |

7. I agree with the placement of "Administer parenteral antibiotics (maternal)" at first line level of care as proposed in the prototype. *

|  | Agree with placement at first line level |
| --- | --- |
|  | Disagree- should be placed at comprehensive level |
|  | Disagree- should be placed at intensive level |

Comments:

|  |
| --- |

8. I agree with the placement of "Administer magnesium sulfate for severe pre-eclampsia or eclampsia" at first line level of care as proposed in the prototype. *

|  | Agree with placement at first line level |
| --- | --- |
|  | Disagree- should be placed at comprehensive level |
|  | Disagree- should be placed at intensive level |

Comments:

|  |
| --- |

9. I agree with the placement of "Administer medications to treat post-partum-haemorrhage (PPH)" at first line level of care as proposed in the prototype. *

|  | Agree with placement at first line level |
| --- | --- |
|  | Disagree- should be placed at comprehensive level |
|  | Disagree- should be placed at intensive level |

Comments:

|  |
| --- |

10. I agree with the placement of "Provide IV fluid replacement therapy (e.g. for shock or sepsis)" at first line level of care as proposed in the prototype. *

|  | Agree with placement at first line level |
| --- | --- |
|  | Disagree- should be placed at comprehensive level |
|  | Disagree- should be placed at intensive level |

Comments:

|  |
| --- |

11. I agree with the placement of "Perform removal of retained products of conception (POC) for abortion or post-abortion care (e.g., vacuum aspiration, dilation and evacuation, medical management)" at the first line level of care as proposed in the prototype. *

|  | Agree with placement at first line level |
| --- | --- |
|  | Disagree- should be placed at comprehensive level |
|  | Disagree- should be placed at intensive level |

Comments:

|  |
| --- |

12. I agree with the placement of "Perform manual removal of retained placenta and uterine exploration" at the comprehensive level of care as proposed in the prototype. *

|  | Agree with placement at comprehensive level |
| --- | --- |
|  | Disagree- should be placed at first line level |
|  | Disagree- should be placed at intensive level |

Comments:

|  |
| --- |

13. I agree with the placement of "Perform assisted vaginal birth (e.g. vacuum extractor, ventouse, forceps)" at the comprehensive level of care as proposed in the prototype. *

|  | Agree with placement at comprehensive level |
| --- | --- |
|  | Disagree- should be placed at first line level |
|  | Disagree- should be placed at intensive level |

Comments:

|  |
| --- |

14. I agree with the placement of "Perform blood transfusion" at the comprehensive level of care as proposed in the prototype. *

|  | Agree with placement at comprehensive level |
| --- | --- |
|  | Disagree- should be placed at first line level |
|  | Disagree- should be placed at intensive level |

Comments:

|  |
| --- |

15. I agree with the placement of "Perform caesarean section" at the comprehensive level of care as proposed in the prototype. *

|  | Agree with placement at comprehensive level |
| --- | --- |
|  | Disagree- should be placed at first line level |
|  | Disagree- should be placed at intensive level |

Comments:

|  |
| --- |

16. I agree with the placement of "Provide intensive level organ support" at the intensive level of care as proposed in the prototype. *

|  | Agree with placement at intensive level |
| --- | --- |
|  | Disagree- should be placed at first line level |
|  | Disagree- should be placed at comprehensive level |

Comments:

|  |
| --- |

6. Newborn

Newborn Care Signal Functions

An ideal EmONC signal function for the revised EmONC framework should meet these criteria: 

CRITICAL: The signal function should represent a clinical intervention or service performed by health workers to manage one or more complication(s) that would otherwise result in major cause/s of maternal or newborn death and disability, or stillbirth.
a TRACER: Performance of the intervention or service ideally indicates that multiple other components of treatment or aspects of care are also present (e.g., performance of surgery generally indicates the availability of anaesthesia).
FREQUENT: The intervention or service should be performed often enough to reflect ongoing performance or functionality of the facility.
SIMPLE: Not necessarily simple to perform, but the intervention or service should be clearly and operationally defined and feasible to measure reliably across contexts.

Emergency obstetric and newborn care (EmONC) signal functions by three levels of care: A prototype

*These signal functions are required at comprehensive and intensive EmONC levels, but they can (and often will) be part of service delivery at first line EmONC facilities.

17. Based on the above criteria, the following newborn signal functions should be included in the revised EmONC framework: *

|  | Agree | Disagree |
| --- | --- | --- |
| Administer antenatal corticosteroids (ACS) to women at risk of preterm birth |  |  |
| Perform neonatal resuscitation with bag and mask |  |  |
| Initiate kangaroo mother care (for LBW/preterm newborns) |  |  |
| Administer oxygen therapy for respiratory support |  |  |
| Administer parenteral antibiotics (newborn) |  |  |
| Perform assisted feeding with expressed breastmilk (e.g. cup and/or nasogastric feeding) |  |  |
| Administer phototherapy for hyperbilirubinemia (jaundice) |  |  |
| Provide thermal care (e.g. with radiant warmer, incubator) |  |  |
| Perform blood transfusion (newborn) |  |  |
| Administer continuous positive airway pressure (CPAP) (newborn) |  |  |
| Administer mechanical ventilation (newborn) |  |  |
| Perform services for retinopathy of prematurity |  |  |
| Provide continued clinical care during interfacility transfer |  |  |

Comments:

|  |
| --- |

18. I agree with the placement of "Provide continued clinical care during interfacility transfer" at the first line level of care as proposed in the prototype. *

|  | Agree with placement at first line level |
| --- | --- |
|  | Disagree- should be placed at comprehensive level |
|  | Disagree- should be placed at intensive level |

Comments:

|  |
| --- |

19. I agree with the placement of "Perform neonatal resuscitation with bag and mask" at the first line level of care as proposed in the prototype. *

|  | Agree with placement at first line level |
| --- | --- |
|  | Disagree- should be placed at comprehensive level |
|  | Disagree- should be placed at intensive level |

Comments:

|  |
| --- |

20. I agree with the placement of "Initiate kangaroo mother care (for LBW/preterm newborns)" at the first line level of care as proposed in the prototype. *

|  | Agree with placement at first line level |
| --- | --- |
|  | Disagree- should be placed at comprehensive level |
|  | Disagree- should be placed at intensive level |

Comments:

|  |
| --- |

21. I agree with the placement of "Perform assisted feeding with expressed breastmilk (e.g. cup and/or nasogastric feeding)" at the first line level of care as proposed in the prototype.*

|  | Agree with placement at first line level |
| --- | --- |
|  | Disagree- should be placed at comprehensive level |
|  | Disagree- should be placed at intensive level |

Comments:

|  |
| --- |

22. I agree with the placement of "Administer oxygen therapy for respiratory support" at the first line level of care as proposed in the prototype. *

|  | Agree with placement at first line level |
| --- | --- |
|  | Disagree- should be placed at comprehensive level |
|  | Disagree- should be placed at intensive level |

Comments:

|  |
| --- |

23. I agree with the placement of "Administer parenteral antibiotics (newborn)" at the first line level of care as proposed in the prototype. *

|  | Agree with placement at first line level |
| --- | --- |
|  | Disagree- should be placed at comprehensive level |
|  | Disagree- should be placed at intensive level |

Comments:

|  |
| --- |

24. I agree with the placement of "Provide thermal care (e.g. with radiant warmer, incubator)" at the comprehensive level of care as proposed in the prototype. *

|  | Agree with placement at comprehensive level |
| --- | --- |
|  | Disagree- should be placed at first line level |
|  | Disagree- should be placed at intensive level |

Comments:

|  |
| --- |

25. I agree with the placement of "Administer antenatal corticosteroids (ACS) to women at risk of preterm birth" at the comprehensive level of care as proposed in the prototype. *

|  | Agree with placement at comprehensive level |
| --- | --- |
|  | Disagree- should be placed at first line level |
|  | Disagree- should be placed at intensive level |

Comments:

|  |
| --- |

26. I agree with the placement of "Administer phototherapy for hyperbilirubinemia (jaundice)" at the comprehensive level of care as proposed in the prototype. *

|  | Agree with placement at comprehensive level |
| --- | --- |
|  | Disagree- should be placed at first line level |
|  | Disagree- should be placed at intensive level |

Comments:

|  |
| --- |

27. I agree with the placement of "Perform services for retinopathy of prematurity" at the comprehensive level of care as proposed in the prototype. *

|  | Agree with placement at comprehensive level |
| --- | --- |
|  | Disagree- should be placed at first line level |
|  | Disagree- should be placed at intensive level |

Comments:

|  |
| --- |

28. I agree with the placement of "Administer continuous positive airway pressure (CPAP) (newborn)" at the comprehensive level of care as proposed in the prototype. *

|  | Agree with placement at comprehensive level |
| --- | --- |
|  | Disagree- should be placed at first line level |
|  | Disagree- should be placed at intensive level |

Comments:

|  |
| --- |

29. I agree with the placement of "Perform blood transfusion (newborn)" at the intensive level of care as proposed in the prototype. *

|  | Agree with placement at intensive level |
| --- | --- |
|  | Disagree- should be placed at first line level |
|  | Disagree- should be placed at comprehensive level |

Comments:

|  |
| --- |

30. I agree with the placement of "Administer mechanical ventilation (newborn)" at the intensive level of care as proposed in the prototype. *

|  | Agree with placement at intensive level |
| --- | --- |
|  | Disagree- should be placed at first line level |
|  | Disagree- should be placed at comprehensive level |

Comments:

|  |
| --- |

7. Obstetric and Newborn

Obstetric care signal functions

An ideal EmONC signal function for the revised EmONC framework should meet these criteria: 

CRITICAL: The signal function should represent a clinical intervention or service performed by health workers to manage one or more complication(s) that would otherwise result in major cause/s of maternal or newborn death and disability, or stillbirth.
a TRACER: Performance of the intervention or service ideally indicates that multiple other components of treatment or aspects of care are also present (e.g., performance of surgery generally indicates the availability of anaesthesia).
FREQUENT: The intervention or service should be performed often enough to reflect ongoing performance or functionality of the facility.
SIMPLE: Not necessarily simple to perform, but the intervention or service should be clearly and operationally defined and feasible to measure reliably across contexts.

Emergency obstetric and newborn care (EmONC) signal functions by three levels of care: A prototype

*These signal functions are required at comprehensive and intensive EmONC levels, but they can (and often will) be part of service delivery at first line EmONC facilities.

31. Based on the above criteria, the following obstetric signal functions should be included in the revised EmONC framework: *

|  | Agree | Disagree |
| --- | --- | --- |
| Administer parenteral antibiotics (maternal) |  |  |
| Administer medications to treat post-partum-haemorrhage (PPH) |  |  |
| Administer magnesium sulfate for severe pre-eclampsia or eclampsia |  |  |
| Provide IV fluid replacement therapy (e.g. for shock or sepsis) |  |  |
| Perform manual removal of retained placenta and uterine exploration |  |  |
| Perform removal of retained products of conception (POC) for abortion or post-abortion care (e.g., vacuum aspiration, dilation and evacuation, medical management) |  |  |
| Perform assisted vaginal birth (e.g. vacuum extractor, ventouse, forceps) |  |  |
| Perform blood transfusion |  |  |
| Perform caesarean section |  |  |
| Provide intensive level organ support |  |  |
| Provide continued clinical care during interfacility transfer |  |  |

Comments:

|  |
| --- |

32. I agree with the placement of "Administer parenteral antibiotics (maternal)" at first line level of care as proposed in the prototype. *

|  | Agree with placement at first line level |
| --- | --- |
|  | Disagree- should be placed at comprehensive level |
|  | Disagree- should be placed at intensive level |

Comments:

|  |
| --- |

33. I agree with the placement of "Administer magnesium sulfate for severe pre-eclampsia or eclampsia" at first line level of care as proposed in the prototype. *

|  | Agree with placement at first line level |
| --- | --- |
|  | Disagree- should be placed at comprehensive level |
|  | Disagree- should be placed at intensive level |

Comments:

|  |
| --- |

34. I agree with the placement of "Administer medications to treat post-partum-haemorrhage (PPH)" at first line level of care as proposed in the prototype. *

|  | Agree with placement at first line level |
| --- | --- |
|  | Disagree- should be placed at comprehensive level |
|  | Disagree- should be placed at intensive level |

Comments:

|  |
| --- |

35. I agree with the placement of "Provide IV fluid replacement therapy (e.g. for shock or sepsis)" at first line level of care as proposed in the prototype. *

|  | Agree with placement at first line level |
| --- | --- |
|  | Disagree- should be placed at comprehensive level |
|  | Disagree- should be placed at intensive level |

Comments:

|  |
| --- |

36. I agree with the placement of "Perform removal of retained products of conception (POC) for abortion or post-abortion care (e.g., vacuum aspiration, dilation and evacuation, medical management)" at the first line level of care as proposed in the prototype. *

|  | Agree with placement at first line level |
| --- | --- |
|  | Disagree- should be placed at comprehensive level |
|  | Disagree- should be placed at intensive level |

Comments:

|  |
| --- |

37. I agree with the placement of "Perform manual removal of retained placenta and uterine exploration" at the comprehensive level of care as proposed in the prototype. *

|  | Agree with placement at comprehensive level |
| --- | --- |
|  | Disagree- should be placed at first line level |
|  | Disagree- should be placed at intensive level |

Comments:

|  |
| --- |

38. I agree with the placement of "Perform assisted vaginal birth (e.g. vacuum extractor, ventouse, forceps)" at the comprehensive level of care as proposed in the prototype. *

|  | Agree with placement at comprehensive level |
| --- | --- |
|  | Disagree- should be placed at first line level |
|  | Disagree- should be placed at intensive level |

Comments:

|  |
| --- |

39. I agree with the placement of "Perform blood transfusion" at the comprehensive level of care as proposed in the prototype. *

|  | Agree with placement at comprehensive level |
| --- | --- |
|  | Disagree- should be placed at first line level |
|  | Disagree- should be placed at intensive level |

Comments:

|  |
| --- |

40. I agree with the placement of "Perform caesarean section" at the comprehensive level of care as proposed in the prototype. *

|  | Agree with placement at comprehensive level |
| --- | --- |
|  | Disagree- should be placed at first line level |
|  | Disagree- should be placed at intensive level |

Comments:

|  |
| --- |

41. I agree with the placement of "Provide intensive level organ support" at the intensive level of care as proposed in the prototype. *

|  | Agree with placement at intensive level |
| --- | --- |
|  | Disagree- should be placed at first line level |
|  | Disagree- should be placed at comprehensive level |

Comments:

|  |
| --- |

Newborn Care Signal Functions

An ideal EmONC signal function for the revised EmONC framework should meet these criteria: 

CRITICAL: The signal function should represent a clinical intervention or service performed by health workers to manage one or more complication(s) that would otherwise result in major cause/s of maternal or newborn death and disability, or stillbirth.
a TRACER: Performance of the intervention or service ideally indicates that multiple other components of treatment or aspects of care are also present (e.g., performance of surgery generally indicates the availability of anaesthesia).
FREQUENT: The intervention or service should be performed often enough to reflect ongoing performance or functionality of the facility.
SIMPLE: Not necessarily simple to perform, but the intervention or service should be clearly and operationally defined and feasible to measure reliably across contexts.

Emergency obstetric and newborn care (EmONC) signal functions by three levels of care: A prototype

*These signal functions are required at comprehensive and intensive EmONC levels, but they can (and often will) be part of service delivery at first line EmONC facilities.

42. Based on the above criteria, the following newborn signal functions should be included in the revised EmONC framework: *

|  | Agree | Disagree |
| --- | --- | --- |
| Administer antenatal corticosteroids (ACS) to women at risk of preterm birth |  |  |
| Perform neonatal resuscitation with bag and mask |  |  |
| Initiate kangaroo mother care (for LBW/preterm newborns) |  |  |
| Administer oxygen therapy for respiratory support |  |  |
| Administer parenteral* antibiotics (newborn) |  |  |
| Perform assisted feeding with expressed breastmilk (e.g. cup and/or nasogastric feeding) |  |  |
| Administer phototherapy for hyperbilirubinemia (jaundice) |  |  |
| Provide thermal care (e.g. with radiant warmer, incubator) |  |  |
| Perform blood transfusion (newborn) |  |  |
| Administer continuous positive airway pressure (CPAP) (newborn) |  |  |
| Administer mechanical ventilation (newborn) |  |  |
| Perform services for retinopathy of prematurity |  |  |
| Provide continued clinical care during interfacility transfer |  |  |

Comments:

|  |
| --- |

43. I agree with the placement of "Provide continued clinical care during interfacility transfer" at the first line level of care as proposed in the prototype. *

|  | Agree with placement at first line level |
| --- | --- |
|  | Disagree- should be placed at comprehensive level |
|  | Disagree- should be placed at intensive level |

Comments:

|  |
| --- |

44. I agree with the placement of "Perform neonatal resuscitation with bag and mask" at the first line level of care as proposed in the prototype. *

|  | Agree with placement at first line level |
| --- | --- |
|  | Disagree- should be placed at comprehensive level |
|  | Disagree- should be placed at intensive level |

Comments:

|  |
| --- |

45. I agree with the placement of "Initiate kangaroo mother care (for LBW/preterm newborns)" at the first line level of care as proposed in the prototype. *

|  | Agree with placement at first line level |
| --- | --- |
|  | Disagree- should be placed at comprehensive level |
|  | Disagree- should be placed at intensive level |

Comments:

|  |
| --- |

46. I agree with the placement of "Perform assisted feeding with expressed breastmilk (e.g. cup and/or nasogastric feeding)" at the first line level of care as proposed in the prototype. *

|  | Agree with placement at first line level |
| --- | --- |
|  | Disagree- should be placed at comprehensive level |
|  | Disagree- should be placed at intensive level |

Comments:

|  |
| --- |

47. I agree with the placement of "Administer oxygen therapy for respiratory support" at the first line level of care as proposed in the prototype. *

|  | Agree with placement at first line level |
| --- | --- |
|  | Disagree- should be placed at comprehensive level |
|  | Disagree- should be placed at intensive level |

Comments:

|  |
| --- |

48. I agree with the placement of "Administer parenteral antibiotics (newborn)" at the first line level of care as proposed in the prototype. *

|  | Agree with placement at first line level |
| --- | --- |
|  | Disagree- should be placed at comprehensive level |
|  | Disagree- should be placed at intensive level |

Comments:

|  |
| --- |

49. I agree with the placement of "Provide thermal care (e.g. with radiant warmer, incubator)" at the comprehensive level of care as proposed in the prototype. *

|  | Agree with placement at comprehensive level |
| --- | --- |
|  | Disagree- should be placed at first line level |
|  | Disagree- should be placed at intensive level |

Comments:

|  |
| --- |

50. I agree with the placement of "Administer antenatal corticosteroids (ACS) to women at risk of preterm birth" at the comprehensive level of care as proposed in the prototype. *

|  | Agree with placement at comprehensive level |
| --- | --- |
|  | Disagree- should be placed at first line level |
|  | Disagree- should be placed at intensive level |

Comments:

|  |
| --- |

51. I agree with the placement of "Administer phototherapy for hyperbilirubinemia (jaundice)" at the comprehensive level of care as proposed in the prototype. *

|  | Agree with placement at comprehensive level |
| --- | --- |
|  | Disagree- should be placed at first line level |
|  | Disagree- should be placed at intensive level |

Comments:

|  |
| --- |

52. I agree with the placement of "Perform services for retinopathy of prematurity" at the comprehensive level of care as proposed in the prototype. *

|  | Agree with placement at comprehensive level |
| --- | --- |
|  | Disagree- should be placed at first line level |
|  | Disagree- should be placed at intensive level |

Comments:

|  |
| --- |

53. I agree with the placement of "Administer continuous positive airway pressure (CPAP) (newborn)" at the comprehensive level of care as proposed in the prototype. *

|  | Agree with placement at comprehensive level |
| --- | --- |
|  | Disagree- should be placed at first line level |
|  | Disagree- should be placed at intensive level |

Comments:

|  |
| --- |

54. I agree with the placement of "Perform blood transfusion (newborn)" at the intensive level of care as proposed in the prototype. *

|  | Agree with placement at intensive level |
| --- | --- |
|  | Disagree- should be placed at first line level |
|  | Disagree- should be placed at comprehensive level |

Comments:

|  |
| --- |

55. I agree with the placement of "Administer mechanical ventilation (newborn)" at the intensive level of care as proposed in the prototype. *

|  | Agree with placement at intensive level |
| --- | --- |
|  | Disagree- should be placed at first line level |
|  | Disagree- should be placed at comprehensive level |

Comments:

|  |
| --- |

**8. Additional questions**

56. Beyond the signal functions included in the prototype, are there signal functions for interventions for preventing stillbirth that you consider meet the signal function criteria?

|  |
| --- |

57. ​​​​​​​Beyond the signal functions included in the prototype, are there signal functions that capture interventions that benefit women and newborns together that you consider meet the signal function criteria?

|  |
| --- |

58. Are there any other signal functions other than those shown on the prototype that you think are essential to include in the revised EmONC framework?

|  |
| --- |

9. Thank You

59. I would like to receive communications and information about the revised EmONC framework and indicators.

|  | Yes |
| --- | --- |
|  | No |

60. I would be interested to provide feedback on other aspects of the EmONC framework and indicators as it develops.

|  | Yes |
| --- | --- |
|  | No |

Thank you for participating in all three rounds of this Delphi study on obstetric and newborn care signal functions and levels of care!

Best regards,
Dr Sarah Moxon, Dr Sudha Sharma and Dr Jalemba Aluvaala
On behalf of the Re-Visioning EmONC Project Steering Committee
