# Supplementary material for: A Delphi process to build consensus on revised Emergency Obstetric and Newborn Care (EmONC) signal functions and levels of care
Source: PLoS One. 2025 Sep 22;20(9):e0331684. doi: 10.1371/journal.pone.0331684 (PMC12453252; doi:10.1371/journal.pone.0331684)
Supplement: S6 Appendix — (DOCX) [file pone.0331684.s006.docx]

**S6 Appendix: R2 summary of results**

**Emergency obstetric signal functions**

| **Historical status** | **Selected by Delphi R2 (yes/no)** | **Signal function** | **Proposed change to wording of signal function from Delphi R2** | **Intervention description** | **Other notes** |
| --- | --- | --- | --- | --- | --- |
| Existing (basic & comprehensive) | **Yes** | Administer parenteral* antibiotics (e.g. ampicillin, gentamicin) (maternal) | Administer *appropriate* parenteral antibiotics (maternal) | Parenteral antibiotics are used for suspected or established severe maternal infection or maternal sepsis (e.g., reproductive tract infection following abortion or childbirth). Their use is also recommended in cases of maternal generalised bacteraemia and septicaemia. | Broad spectrum antibiotics often in use – should not specify gentamicin and ampicillin. Metronidazole often used.  Concerns about misuse/overuse of antibiotics in many settings. |
| Existing (basic & comprehensive) | **Yes** | Administer appropriate medications to treat post-partum haemorrhage (PPH) (appropriate medications in the algorithm for PPH e.g. oxytocin or ergometrine (or combination of oxytocin and ergometrine), or oral misoprostol, or prostaglandin or heat stable carbetocin or tranexamic acid). | *Administer appropriate medications to treat post-partum haemorrhage (PPH) including uterotonic and tranexamic acid* | Uterotonics (oxytocin alone as the first choice) are used to treat PPH most commonly caused by atonic uterus. They can also be used to treat bleeding after an incomplete late abortion along with other definitive treatment. In settings where oxytocin is unavailable, the use of other injectable uterotonics (if appropriate ergometrine or the fixed drug combination of oxytocin and ergometrine) or oral misoprostol is recommended. The use of tranexamic acid is recommended for the treatment of PPH if oxytocin and other uterotonics fail to stop bleeding or if it is thought that the bleeding may be partly due to trauma. Heat stable carbetocin can also be used in certain contexts. | Concerns that this was too wordy, confusing, and composite with some issues with the proposed algorithm of drugs.  Note that heat stable carbetocin (HSC) not used for treatment. TXA should be used in all cases of PPH. Many suggestions for simplification.  ?Remove mention of heat stable carbetocin. |
| Existing (basic & comprehensive) | **Yes** | Administer magnesium sulfate for pre-eclampsia or eclampsia | Administer magnesium sulfate for *severe* pre-eclampsia or eclampsia | Magnesium sulfate is given to women diagnosed with severe pre-eclampsia or eclampsia to prevent and treat convulsions. | Suggestions that severe should be added as a qualifier to the signal function |
| **New** | **Yes** | Administer maternal antihypertensives (e.g. alpha methyldopa, hydralazine, labetalol, nifedipine) |  | Antihypertensives are used to manage both gestational and chronic hypertension. For women diagnosed with severe pre-eclampsia or eclampsia where blood pressure remains high, antihypertensives are administered by IV route, orally or sublingually. | Comments that this is not an emergency intervention – need further clarification on what anti-hypertensive route and situation.  Comments also to combine magneisum sulfate and anti-hypertensives (in the same way that PPH medications are mixed). |
| **New** | **Yes** | Provide intravenous (IV) infusion/IV fluid replacement therapy (e.g. maternal resuscitation) |  | Intravenous replacement fluids *during pregnancy and* labour as replacement therapy or in the postpartum period to treat shock are the first-line treatment for hypovolaemia due to haemorrhage (antepartum/post-partum), sepsis or other cause. In case of post-partum haemorrhage it maintains the circulation whilst interventions to control bleeding are performed and assessment for whether blood transfusion is needed. | Comments that this is difficult to frame as an indicator or signal function without sufficient explanation to differentiate between routine administration of IV fluids and IV fluids for treatment of shock. Also respondents notes that if a facility is administering parenteral antibiotics or blood transfusion it can also provide IV fluid for maternal resuscitation. |
| Existing (basic & comprehensive) | **Yes** | Perform manual uterine exploration and removal of placenta | Perform manual uterine exploration and removal of placenta *for retained placenta* | This procedure addresses retained placenta or fragments of the placenta. It entails full antiseptic procedure, pain relief, and insertion of gloved hand into the uterus to locate the edge of the placenta, separating the placenta in its entirety from the uterine wall and removing it. | Requests to specify under GA/analgesia and to specify that the procedure is for retained placenta. This is often confused with removal or retained products therefore needs to be more specific. |
| Existing (basic & comprehensive) | **Yes** | Perform removal of retained products of conception (POC) (e.g. manual vacuum aspiration (MVA), dilation and curettage (D&C), medical management) | Perform removal of retained products of conception (POC) (e.g. manual vacuum aspiration (MVA), dilation and curettage (D&C), medical management) *for post-abortion care*  *OR*  *Manage post-abortal complications through removal of retained products of conception (POC) (e.g. manual vacuum aspiration (MVA), dilation and curettage (D&C), medical management)* | Retained products of conception (POC) can occur as a result of an incomplete spontaneous or induced abortion. MVA procedure entails inserting an appropriate sized cannula into the uterus, creating vacuum in a plastic syringe, connecting it to the cannula and removing the POC with gentle aspiration using a rotating movement. POC can also be removed by dilatation of cervix and curettage with a sharp curette but MVA is a comparatively safer procedure. | Comments here to be more explicit that this refers to post-abortion care.  Is there a need to integrate abortifacient medical products to the signal function. |
| Existing (basic & comprehensive) | **No** | Perform assisted vaginal birth (e.g. vacuum extractor, forceps) |  | This procedure is performed during the second stage of labour to deliver the baby for indications of fetal distress or prolonged second stage of labour. Prerequisite conditions are a skilled health provider, the cervix is fully dilated and fetal head has descended sufficiently. | Suggestion to remove altogether – often confused with attended delivery and confusion with French language.  Forceps delivery is not practiced except in exceptional cases at the tertiary level. Vacuum cup is important but rarely used for several reasons.  Suggestions to add Kiwi type vacuum. |
| Existing (comprehensive) | **Yes** | Perform blood transfusion |  | The need for the transfusion of whole blood or blood products can occur due to antepartum or postpartum haemorrhage leading to shock, loss of a large volume of blood such as with ruptured ectopic pregnancy or at operative birth, coagulation disorders or severe anaemia late in pregnancy. | Suggestion that could also add substitutes/blood replacement. |
| Existing (comprehensive) | **Yes** | Perform caesarean section | Perform *emergency* caesarean section  OR  *Perform surgery including caesarean section and exploratory laparotomy* | Caesarean section is performed for multiple maternal and fetal indications that commonly include prolonged or obstructed labour, fetal distress, malpresentation or placenta previa. It is assumed that if caesarean section is performed the facility is also capable of providing anaesthesia. | Concerns about being clear on the use of c-section for obstetric indications (avoiding misuse)  Do not want to miss out the management of ectopic pregnancy. |
| **New** | **No** | Perform mechanical ventilation | No suggestions | Assisted ventilation may be needed in cases of respiratory failure due to severe life threatening condition/s during pregnancy or in the postpartum period. This is usually provided in the intensive care unit along with other supportive measures. | Needs to be more clearly defined if to be included. |
| **New** | **No** | Provide high dependency/intensive-care | No suggestions | Critically ill pregnant women need multi-organ support to prevent mortality and morbidity. This is best provided in a high-dependency/ intensive care unit with nurses/midwives and doctors with intensive care skills. | Needs to be more clearly defined if to be included. |

* Parenteral includes administration of a drug through the intravenous route or as intra-muscular injections.

**Content above has been adapted from:**

World Health Organization, UNFPA, UNICEF. *Managing Complications in Pregnancy and Childbirth: a guide for midwives and doctors* – 2^nd^ edition, Geneva: World Health Organization; 2017. Licence: CC BY-NC-SA 3.0 IGO.

**Referral signal function for obstetric and newborn care**

| **Historical status** | **Selected by Delphi R2 (yes/no)** | **Signal function** | **Proposed change to wording of signal function from Delphi R2** | **Intervention description** | **Other notes** |
| --- | --- | --- | --- | --- | --- |
| **New** | **No** | Provision of continued emergency clinical obstetric and newborn care during interfacility transfer | *Provision of continued clinical care during interfacility transfer*  *OR*  *Coordinated transport to a higher level facility* | Continued emergency clinical obstetric and neonatal care during interfacility transfer refers to transport of obstetric and newborn patients with a clinically trained health worker and adequate equipment to ensure ongoing provision of emergency care during transfer between facilities. | Would rely on improved documentation to make measurement more feasible.  Concerns that this is not simple enough for a signal function.  Necessary to specify what care for this function and by whom for it to be effective.  Needs more refining e.g. in vehicle equipped to support continuous oxygen, fluids by accompanying HW (otherwise could be a health worker in the back of a taxi). |

*** Parenteral includes administration of a drug through the intravenous route or as intra-muscular injections.**

**Small and sick newborn signal functions**

| **Historical**  **status** | **Selected by Delphi R2 (yes/no)** | **Signal function** | **Proposed change to wording of signal function** | **Intervention description/definition** | **Other notes** |
| --- | --- | --- | --- | --- | --- |
| **New** | **Yes** | Administer antenatal corticosteroids (ACS) (e.g. dexamethasone, betamethasone) to women at risk of preterm birth | Administer *complete course* of antenatal corticosteroids to women at risk of preterm birth | The administration of ACS to women at risk of imminent preterm birth is used to stimulate fetal lung maturation and refers to dexamethasone or betamethasone administered by intramuscular injection for women at risk of imminent preterm birth (anticipated with the subsequent 7 days) from 24 weeks to 34 weeks gestation according to WHO guidelines. The recommendation is that ACS is only offered at a level where resuscitation, thermal care, feeding support, infection treatment and safe oxygen are available. ACS are contraindicated for women with chorioamnionitis. | Administered to woman on labour and delivery ward (not to newborn) therefore should be included as part of obstetric signal functions. |
| **New** | **Yes** | Administer magnesium sulfate to women at risk of preterm birth |  | The use of magnesium sulfate is recommended for women at risk of imminent preterm birth before 32 weeks of gestation for prevention of cerebral palsy in the infant and child. Magnesium sulfate for neuroprotection is recommended if preterm birth is likely within the next 24 hours. | Same as above comment due to timing of intervention. |
| **Existing**  **(basic & compre-**  **hensive)** | **Yes** | Perform neonatal resuscitation with bag and mask |  | Basic newborn resuscitation at the time of birth comprises of a set of interventions required to establish breathing and circulation in a newborn who is not spontaneously breathing/crying at birth. This procedure includes providing positive-pressure ventilation with a bag and mask equipment of an appropriate size (i.e. not referring to simple stimulation by rubbing, drying). | Comment that this should include all steps of resuscitation. |
| **New** | **Yes** | Initiate kangaroo mother care (for LBW/preterm newborns) | *Provide at least 3 days continuous KMC*  *OR*  *Initiate KMC or other thermal care (for LBW/preterm newborns)*  *OR*  Initiate *immediate* kangaroo mother care (for LBW/preterm newborns) | Kangaroo mother care is an approach to care of preterm and/or LBW infants. The signal function refers to initiation of newborns held in the kangaroo position most of the day whereby the infant is placed and held in direct skin-to-skin contact on the mother’s (or other caregiver’s) chest in an up-right position. The aim is for early initiation of KMC and for continuous performance (>18 hours per day). Other key components of KMC are support for exclusive and early breastmilk provision and timely discharge from the hospital with appropriate follow-up. When babies are not in KMC position with mother or other care-giver appropriate thermal care should be provided. | Difficult to prioritise between KMC and radiant warmer as am emergency intervention (with KMC for non-stable babies still not mainstream).  Should be separated by:  -Hemodynamically stable – KMC  -Hemodynamically unstable – radiant. warmer/incubator/heated cot |
| **New** | **Yes** | Administer oxygen therapy with pulse oximetry | Administer oxygen therapy with pulse oximetry *for respiratory support*  *Administer oxygen safely when necessary, with regular monitoring*  *OR*  *Administer oxygen therapy and check oxygen saturation with pulse oximetry* | Small and sick newborns with hypoxia require appropriate oxygen therapy. Pulse oximetry determines the presence of hypoxia and hyperoxia, and guides safe administration of oxygen therapy which involves administration of oxygen via neonatal nasal prong using low-flow metres, air oxygen blenders and humidifiers. | Oxygen is important - but it needs to be delivered in a cautiously and safely for a baby for whom it is deemed necessary.  Concerns about linking pulse oximetry to oxygen as may give false security that high saturations are better. |
| **New** | **Yes** | Administer parenteral* antibiotics (e.g. gentamicin, ampicillin) (newborn) |  | Parenteral antibiotics are used for suspected or established infections including the clinical syndromes of sepsis, meningitis or pneumonia in newborns based on clinical presentation diagnosed via clinical algorithm or confirmed via positive blood culture. |  |
| **New** | **Yes** | Perform assisted feeding with expressed breastmilk (e.g. cup and/or nasogastric feeding) | Perform assisted feeding with expressed breastmilk *or donor milk* (e.g. cup and/or nasogastric feeding) | Assisted feeding of newborns is performed for babies that may not be able to effectively breastfeed due to prematurity, small size or sickness and refers to the provision of cup and/or gastric tube feeding of newborns using expressed breastmilk. | Should include donor milk here  Concerns that this function should also focus on direct breast feeding. |
| **New** | **Yes** | Administer phototherapy for hyperbilirubinemia (jaundice) | Administer phototherapy for *measured* hyperbilirubinaemia | Jaundice is common in all newborns and without treatment can lead to severe illness (e.g. kernicterus) and death. Administering phototherapy for neonatal hyperbilirubinemia refers to treatment with effective, safe phototherapy e.g. high-intensity light emitting diodes (LED). |  |
| **New** | **No** | Administer parenteral* anticonvulsants for seizures (newborn) (e.g. phenobarbitone) |  | Neonatal seizures are some of the most frequent neurological events in newborns, reflecting a variety of pre-, peri- or postnatal disorders of the central nervous system. After excluding hypoglycaemia, administering parenteral anticonvulsants refers to the management of seizures in newborns using phenobarbitone as the first-line medication. |  |
| **New** | **No** | Perform blood transfusion (newborn) |  | The need for transfusion of blood or blood products can occur due to multiple complications including anaemia of prematurity, post-jaundice management or other conditions. Blood transfusion for newborns requires fresh, irradiated blood that is negative for cytomegalovirus and warmed for administration with management of the newborn for transfusion reactions. |  |
| **New** | **No** | Administer CPAP (newborn) | *Initiate and manage* CPAP *for respiratory support* | Continuous positive airway pressure is a non-invasive type of respiratory support which can be delivered without endotracheal intubation and is used for small and sick newborns (especially preterm) with surfactant deficiency. |  |
| **New** | **No** | Perform services for retinopathy of prematurity (ROP) | *Screen for neonatal retinopathy of prematurity (ROP)* | Retinopathy of prematurity, a complication of preterm birth, is a vision threatening disease associated with abnormal retinal vascular development that can lead to blindness and visual impairment. Services for retinopathy of prematurity require inpatient screening of eligible at-risk infants for ROP by trained technician or ophthalmologist. Infants identified as requiring treatment will require laser treatment by indirect delivery or intravitreal injection of AntiVEGF agents for infants developing signs of sight threatening (Type 1) ROP. At most secondary level facilities only screening will be possible (by trained technicians or ophthalmologist) and infants will require a referral to an appropriate centre for treatment. | Services could cause some confusion as to whether this is screening or treatment or prevention. |
| **New** | **No** | Administer intravenous (IV) fluids (newborn) |  | Intravenous fluids (containing glucose) are required for some newborns with delayed or impaired sucking, or those with severe illness. Following WHO guidelines, fluids are administered through an infusion pump and a neonatal burette, the volume is recorded, and the IV site is check regularly. | Could IV fluids, blood transfusion be combined? (this would make a composite signal function). |
| **New** | **Yes** | Provide thermal care (e.g. radiant warmer, incubator, heated cot) for preterm/LBW newborns | *Provide thermoregulation using a functional radiant warmer/incubator* | Preterm newborns and/or LBW infants at risk of hypothermia, who are unstable or who cannot be cared for in kangaroo mother care, require thermal care using a clean incubator or under radiant warmer. Close temperature monitoring is also required. | Some thermal care can be done at lower levels but incubator should not. Should change wording of signal function so that this can be provided at a lower level of care. |
| **New** | **No** | Administer methylxanthines (e.g. caffeine) for preterm newborns for prevention and treatment of apnoea |  | Recurrent apnoea is common in preterm infants. Methylxanthines (caffeine as the preferred drug) are used for preterm newborns <32 weeks to stimulate breathing and reduce apnoea and its consequences. |  |

*Parenteral includes administration of a drug through the intravenous route or as intra-muscular injections.

**Content above has been adapted from:**

World Health Organization, UNFPA, UNICEF (2017). *Managing Complications in Pregnancy and Childbirth: a guide for midwives and doctors* – 2^nd^ edition, Geneva: World Health Organization

World Health Organization (2020). *Standards for improving quality of care for small and sick newborns in health facilities*. Geneva: World Health Organization

World Health Organization (2015) *World Health Organization recommendations on interventions to improve preterm birth outcomes*. Geneva: World Health Organization.

**R2 Average rank score obstetric signal functions**

| **Rank** | **Signal function** | **Average rank** |
| --- | --- | --- |
| 1 | Administer appropriate medications to treat post-partum haemorrhage (PPH) (appropriate medications in the algorithm for PPH e.g. oxytocin or ergometrine (or combination of oxytocin and ergometrine), or oral misoprostol, or prostaglandin or heat stable carbetocin or tranexamic acid). | 1.96 |
| 2 | Administer magnesium sulfate for pre-eclampsia or eclampsia | 3.39 |
| 3 | Administer parenteral antibiotics (e.g. ampicillin, gentamicin) (maternal) | 3.94 |
| 4 | Perform manual uterine exploration and removal of placenta | 6.51 |
| 5 | Administer maternal antihypertensives (e.g. alpha methyldopa, hydralazine, labetalol, nifedipine) | 6.70 |
| 6 | Provide intravenous (IV) infusion/IV fluid replacement therapy (e.g. maternal resuscitation) | 6.73 |
| 7 | Perform removal of retained products of conception (e.g. manual vacuum aspiration), dilation and curettage, medical management) | 6.78 |
| 8 | Perform caesarean section | 7.07 |
| 9 | Perform blood transfusion | 7.18 |
| 10 | Perform assisted vaginal birth (e.g., vacuum extractor, forceps) | 7.98 |
| 11 | Provision of continued emergency clinical obstetric and newborn care during interfacility transfer | 9.72 |
| 12 | Perform mechanical ventilation | 11.31 |
| 13 | Provide high dependency/intensive care | 11.72 |

**R2 Average rank scores newborn signal functions**

| **Rank** | **Signal function** | **Average rank** |
| --- | --- | --- |
| 1 | Perform neonatal resuscitation with bag and mask | 1.79 |
| 2 | Initiate kangaroo mother care (for LBW/preterm newborns) | 4.91 |
| 3 | Administer antenatal corticosteroids (ACS) (e.g. dexamethasone, betamethasone) to women at risk of preterm birth | 5.33 |
| 4 | Administer parenteral antibiotics (e.g. gentamicin, ampicillin) (newborn) | 5.36 |
| 5 | Administer oxygen therapy with pulse oximetry | 5.54 |
| 6 | Perform assisted feeding with expressed breastmilk (e.g. cup and/or nasogastric feeding) | 7.47 |
| 7 | Administer magnesium sulfate to women at risk of preterm birth | 8.21 |
| 8 | Administer phototherapy for hyperbilirubinemia (jaundice) | 8.93 |
| 9 | Provide thermal care (e.g. radiant warmer, incubator, heated cot) for preterm/LBW newborns | 9.04 |
| 10 | Administer CPAP (newborn) | 9.11 |
| 11 | Administer parenteral anticonvulsants for seizures (newborn) (e.g. phenobarbitone) | 9.82 |
| 12 | Administer intravenous (IV) fluids (newborn) | 10.64 |
| 13 | Provision of continued emergency clinical obstetric and newborn care during interfacility transfer | 11.13 |
| 14 | Perform blood transfusion (newborn) | 11.83 |
| 15 | Perform services for retinopathy of prematurity | 13.26 |
| 16 | Administer methylxanthines (e.g. caffeine) for preterm newborns for prevention and treatment of apnoea | 13.62 |
