# Supplementary material for: A Delphi process to build consensus on revised Emergency Obstetric and Newborn Care (EmONC) signal functions and levels of care
Source: PLoS One. 2025 Sep 22;20(9):e0331684. doi: 10.1371/journal.pone.0331684 (PMC12453252; doi:10.1371/journal.pone.0331684)
Supplement: S7 Appendix — (DOCX) [file pone.0331684.s007.docx]

**S7 Appendix. R3 free text summaries on EmONC signal functions**

**Obstetric signal functions**

| **Historical status** | **Signal function** | **Proposed change to wording of signal function from Delphi R3** | **Intervention description** | **Other notes/changes proposed** |
| --- | --- | --- | --- | --- |
| Existing (basic & comprehensive) | **Administer parenteral* antibiotics (maternal)** | No specific proposed changes | Parenteral antibiotics are used for suspected or established severe maternal infection or maternal sepsis (e.g., reproductive tract infection following abortion or childbirth). Their use is also recommended in cases of maternal generalised bacteraemia and septicaemia. | Often confusion over meaning of parenteral |
| Existing (basic & comprehensive) | **Administer medications to treat post-partum-haemorrhage (PPH)** | No specific proposed changes (see notes) | Uterotonics (oxytocin alone as the first choice) are used to treat PPH most commonly caused by atonic uterus. They can also be used to treat bleeding after an incomplete late abortion along with other definitive treatment. In settings where oxytocin is unavailable, the use of other injectable uterotonics or oral misoprostol is recommended. In addition to other uterotonics, the use of tranexamic acid is recommended for the treatment of PPH if it is thought that the bleeding may be partly due to trauma. | Concerns about the different medications in the algorithm and tracking their use separately.  Suggestions that Anti-shock garment (NASG) could be included. |
| Existing (basic & comprehensive) | **Administer magnesium sulfate for severe pre-eclampsia or eclampsia** | No specific proposed changes | Magnesium sulfate is given to women diagnosed with severe pre-eclampsia or eclampsia to prevent and treat convulsions. | Some concerns that other drugs other than magnesium sulfate may be used for the treatment of the condition. |
| **New** | **Provide IV fluid replacement therapy (e.g. for shock or sepsis)** | No specific proposed changes | Intravenous replacement fluids during pregnancy and labour as replacement therapy or in the postpartum period to treat shock are the first-line treatment for hypovolaemia due to haemorrhage (antepartum/post-partum), sepsis or other cause. In case of post-partum haemorrhage it maintains the circulation whilst interventions to control bleeding are performed and assessment for whether blood transfusion is needed. | IV fluids are available in most health facilities – some concerns that this is not needed as a specific signal function. |
| Existing (basic & comprehensive) | **Perform manual removal of retained placenta and uterine exploration** | No specific proposed changes. | This procedure addresses retained placenta or fragments of the placenta. It entails full antiseptic procedure, pain relief, and insertion of gloved hand into the uterus to locate the edge of the placenta, separating the placenta in its entirety from the uterine wall and removing it. | Ensure there is minimal confusion between MVA and manual removal of placenta |
| Existing (basic & comprehensive) | **Perform removal of retained products of conception (POC) for abortion or post-abortion care (e.g., vacuum aspiration, dilation and evacuation, medical management)** | No specific proposed changes | Retained POC can occur as a result of an incomplete spontaneous or induced abortion. Vacuum aspiration entails inserting an appropriate sized cannula into the uterus, creating vacuum in a plastic syringe, connecting it to the cannula and removing the POC with gentle aspiration using a rotating movement.  POC can also be removed by dilatation and evacuation. | Concerns about D&E at the lowest level and the mix of surgical and medical methods included in the signal function. |
| Existing (basic & comprehensive) | **Perform assisted vaginal birth (e.g. vacuum extractor, ventouse, forceps)** | No specific proposed changes | This procedure is performed during the second stage of labour to deliver the baby for indications of fetal distress or prolonged second stage of labour. Prerequisite conditions are a skilled health provider, fully dilated cervix and a fetal head that has descended sufficiently. |  |
| Existing (comprehensive) | **Perform blood transfusion** | No specific proposed changes | The need for the transfusion of whole blood or blood products can occur due to antepartum or postpartum haemorrhage leading to shock, loss of a large volume of blood such as with ruptured ectopic pregnancy or an operative birth, or with coagulation disorders or severe anaemia late in pregnancy. |  |
| Existing (comprehensive) | **Perform caesarean section** | Some suggestions to revert to include other surgery as well | Caesarean section is performed for multiple maternal and fetal indications that commonly include prolonged or obstructed labour, fetal distress, malpresentation or placenta previa. It is assumed that if caesarean section is performed the facility is also capable of providing anaesthesia. | Qualification or benchmarking may be needed to monitor overuse.  Concerns also that anaesthesia/pain relief should not be assumed to be available and correctly used.  Concerns also that laparotomy and other types of surgery may need to be measured. |
| **New** | **Provide intensive level organ support** | No specific proposed changes but concerns about definition (see other notes) | Intensive-level support of one or more organs is required for women with severe complications, including severe PPH or sepsis, septic shock, acute renal failure or cerebral haemorrhage. | **Needs refinement and better definition – concerns that the signal function is too vague and specific examples needed.** |

* Parenteral includes administration of a drug through the intravenous route or as intra-muscular injections.

Content above has been adapted from:

World Health Organization, UNFPA, UNICEF. *Managing Complications in Pregnancy and Childbirth: a guide for midwives and doctors* – 2^nd^ edition, Geneva: World Health Organization; 2017. Licence: CC BY-NC-SA 3.0 IGO.

**Newborn signal functions**

| **Historical status** | **Signal function** | **Proposed change to wording of signal function from Delphi R3** | **Intervention description/definition** | **Other notes** |
| --- | --- | --- | --- | --- |
| **New** | **Administer antenatal corticosteroids (ACS) to women at risk of preterm birth** | No specific proposed changes | The administration of ACS to women at risk of imminent preterm birth is used to stimulate fetal lung maturation and refers to dexamethasone or betamethasone administered by intramuscular injection for women at risk of imminent preterm birth (anticipated with the subsequent 7 days) from 24 weeks to 34 weeks gestation according to WHO guidelines. The recommendation is that ACS is only offered at a level where resuscitation, thermal care, feeding support, infection treatment and safe oxygen are available. ACS are contraindicated for women with chorioamnionitis. | Proposals to place this with obstetric signal functions given timing of care.  Essential to adhere to WHO safety guidelines. |
| **Existing (basic & comprehensive)** | **Perform neonatal resuscitation with bag and mask** | No specific proposed changes | Basic newborn resuscitation at the time of birth comprises of a set of interventions required to establish breathing and circulation in a newborn who is not spontaneously breathing/crying at birth. This procedure includes providing positive-pressure ventilation with a bag and mask equipment of an appropriate size (i.e. not referring to simple stimulation by rubbing, drying). |  |
| **New** | **Initiate kangaroo mother care (for LBW/preterm newborns)** | **Initiate Perform kangaroo mother care (for LBW/preterm newborns)**  OR  **Perform immediate kangaroo mother care (for LBW/preterm newborns** | Kangaroo mother care is an approach to care of preterm and/or LBW infants. The signal function at the first line EmONC level refers to initiation of newborns held in the kangaroo position most of the day whereby the infant is placed and held in direct skin-to-skin contact on the mother’s (or other caregiver’s) chest in an up-right position. The aim is for early initiation of KMC and for continuous performance (>18 hours per day). KMC provided at a comprehensive or intensive care level would be provided alongside other supportive care as per WHO guidelines. Other key components of KMC are support for exclusive and early breastmilk provision and timely discharge from the hospital with appropriate follow-up. When babies are not in KMC position with the mother or another care-giver, appropriate thermal care should be provided. | Some concerns that this doesn’t fit criteria for signal functions due to the multiple components/dimensions of the intervention and that it requires more complex indicator construction (e.g. composite)  There are known measurement challenges with KMC that need to be explored further. |
| **New** | **Administer oxygen therapy for respiratory support** | No specific proposed changes | Small and sick newborns with hypoxia require appropriate oxygen therapy. At the first line level, safe oxygen therapy may be initiated. At a comprehensive level, it would be expected that safe oxygen therapy is provided, as per small and sick newborn care guidelines. Pulse oximetry determines the presence of hypoxia and hyperoxia, and guides safe administration of oxygen therapy. At a comprehensive and intensive care level, oxygen therapy would involve administration of oxygen via neonatal nasal prong using low-flow metres with pulse oximetry, air oxygen blenders and humidifiers, which are a requirement for oxygen therapy on a neonatal unit as per *WHO small and sick newborn care guidelines.* | Safety concerns and need for monitoring with pulse oximetry.  Many lower level facilities do not have oxygen. |
| **New** | **Administer parenteral* antibiotics (newborn)** | No specific proposed changes | Parenteral antibiotics are used for suspected or established infections including the clinical syndromes of sepsis, meningitis or pneumonia in newborns based on clinical presentation diagnosed via clinical algorithm or confirmed via positive blood culture. At the first line level, antibiotics may be given at least as an intramuscular dose as pre-referral treatment as per WHO guidelines. At a comprehensive and intensive care level, antibiotics may be given as IV doses or infusions as per *WHO small and sick newborn care guidelines.* | Often confusion over meaning of parenteral.  Whilst the lowest level could give the first dose/pre-referral or IM antibiotics, a baby who needs inpatient care and antibiotics would need to be in a comprehensive or level 2 inpatient care facility.  Concerns over antibiotic stewardship. |
| **New** | **Perform assisted feeding with expressed breastmilk (e.g. cup and/or nasogastric feeding)** | No specific proposed changes | Assisted feeding of newborns is performed for babies that may not be able to effectively breastfeed due to prematurity, small size or sickness and refers to the provision of cup and/or gastric tube feeding of newborns using expressed breastmilk or donor milk. | Some concerns that nasogastric tube feeding not appropriate at the lowest level. |
| **New** | **Administer phototherapy for hyperbilirubinemia (jaundice)** | No specific proposed changes | Jaundice is common in all newborns and without treatment can lead to severe illness (e.g. kernicterus) and death. Administering phototherapy for neonatal hyperbilirubinemia refers to treatment with effective, safe phototherapy e.g. high-intensity light emitting diodes (LED). | Bilirubin testing needed as part of care package for jaundice. |
| **New** | **Provide thermal care (e.g. with radiant warmer, incubator)** | No specific proposed changes | Preterm newborns and/or LBW infants at risk of hypothermia who are unstable or who cannot be cared for in kangaroo mother care, require thermal care using a clean incubator or under radiant warmer (or equivalent warming device). Close temperature monitoring is also required. | Concerns that thermal care is part of care for every baby starting with drying and skin to skin at birth – some concerns that it doesn’t fit criteria for a signal function and overlaps or has potential to undermine KMC at comprehensive level. Requires necessary infrastructure (e.g., is about power supply) and maintenance of equipment and supplies and may be better measured as an indicator.  Concerns that this undermines KMC and implies that thermal care is superior or a higher level intervention than KMC. |
| **New** | **Perform blood transfusion (newborn)** | No specific proposed changes | The need for transfusion of blood or blood products can occur due to multiple conditions. Blood transfusion for newborns should be performed with fresh, irradiated blood that is negative for cytomegalovirus and warmed for administration with close monitoring of the newborn for transfusion reactions. | Clarification on whether inclusive of exchange transfusion |
| **New** | **Administer CPAP (newborn)** | No specific proposed changes | Continuous positive airway pressure is a non-invasive type of respiratory support which can be delivered without endotracheal intubation and is used for small and sick newborns (especially preterm) with surfactant deficiency. When administering oxygen via CPAP, attention to safe oxygen therapy as per small and sick newborn guidelines, including use of pulse oximetry at the comprehensive and intensive care level. | This is a key part of ENAP coverage target and is not possible to get to SDG 3.2 of NMR <12 per 1000 live births without respiratory support for preterm so CPAP needs to be widely (and effectively, safely) available |
| **New** | **Administer mechanical ventilation (newborn)** | No specific proposed changes but see other notes. | Mechanical ventilation is an invasive type of respiratory support for extremely small and sick newborns involving endotracheal intubation and breathing support with a mechanical ventilator. Mechanical ventilation requires high level of monitoring and supportive care as per *WHO small and sick newborn guidelines.* | Safety concerns about this as a signal function and concerns about increasing inappropriate use (where CPAP would be more appropriate.  Would mention of surfactant also make sense?  Some suggestions to align more with obstetric intensive level signal function |
| **New** | **Perform services for retinopathy of prematurity** | Care for retinopathy of prematurity | Retinopathy of prematurity, a complication of preterm birth, is a vision-threatening disease associated with abnormal retinal vascular development that can lead to blindness and visual impairment. Services for retinopathy of prematurity require inpatient screening of eligible at-risk infants for ROP by trained technician or ophthalmologist. Infants identified as requiring treatment will require laser treatment by indirect delivery or intravitreal injection of AntiVEGF agents for infants developing signs of sight threatening (Type 1) ROP. At most secondary level facilities only screening will be possible (by trained technicians or ophthalmologist) and infants will require a referral to an appropriate centre for treatment. | Concerns that this doesn’t fit criteria for signal function  Too vague and very setting dependent  Only screening often available at level 2/comprehensive.  Many suggest that services to diagnose would be available at level 2 and that services to treat would only be available at intensive level. |

*Parenteral includes administration of a drug through the intravenous route or as intra-muscular injections.

Content above has been adapted from:

World Health Organization, UNFPA, UNICEF (2017). *Managing Complications in Pregnancy and Childbirth: a guide for midwives and doctors* – 2^nd^ edition, Geneva: World Health Organization

World Health Organization (2020). *Standards for improving quality of care for small and sick newborns in health facilities*. Geneva: World Health Organization

World Health Organization (2015) *WHO recommendations on interventions to improve preterm birth outcomes*. Geneva: World Health Organization.

World Health Organization (2022) *WHO recommendations on antenatal corticosteroids for improving preterm birth outcomes. Geneva:* World Health Organization.

**Referral signal function for obstetric and newborn care**

| **Historical Status** | **Signal function** | **Proposed changes to wording of signal function from Delphi R3** | **Intervention description/definition** | **Other notes** |
| --- | --- | --- | --- | --- |
| **New** | **Provide continued clinical care during interfacility transfer** | Suggestions that it could be re-phrased to include or emphasise *pre-transfer stabilisation* | Continued care during interfacility transfer refers to transport of obstetric and/or newborn patients with a clinically trained health worker and adequate equipment to ensure ongoing provision of emergency care during transfer between facilities. | **Requires further definition and discussion**. Concerns that criteria are very challenging to achieve for many countries. |
