# Supplementary material for: A Delphi process to build consensus on revised Emergency Obstetric and Newborn Care (EmONC) signal functions and levels of care
Source: PLoS One. 2025 Sep 22;20(9):e0331684. doi: 10.1371/journal.pone.0331684 (PMC12453252; doi:10.1371/journal.pone.0331684)
Supplement: Attachment — Submitted filename: Peer review responses_4AUG_FINAL.docx [file pone.0331684.s009.docx]

Delphi study on emergency obstetric and newborn care signal functions

PLOS ONE peer review responses to editor and reviewers

| **PLOS ONE editor comments** | Response/action |
| --- | --- |
| Please ensure that your manuscript meets PLOS ONE's style requirements, including those for file naming. The PLOS ONE style templates can be found at  [https://journals.plos.org/plosone/s/file?id=wjVg/PLOSOne_formatting_sample_main_body.pdf](https://url.uk.m.mimecastprotect.com/s/4eIuCk714TvvY5QUQi3hGymV4?domain=journals.plos.org)  [https://journals.plos.org/plosone/s/file?id=ba62/PLOSOne_formatting_sample_title_authors_affiliations.pdf](https://url.uk.m.mimecastprotect.com/s/Qr2dCl714TllOXZfqsDhzqxex?domain=journals.plos.org) | To the best of our knowledge, the manuscript meets PLOS ONE’s style requirements. All files have been named according to the requirements. |
| Thank you for stating the following financial disclosure: “This work was supported, in whole or in part, by the Bill & Melinda Gates Foundation [INV-001363]. Under the grant conditions of the Foundation, a Creative Commons Attribution 4.0 Generic License has already been assigned to the Author Accepted Manuscript version that might arise from this submission.”  Please state what role the funders took in the study.  If the funders had no role, please state: "The funders had no role in study design, data collection and analysis, decision to publish, or preparation of the manuscript." If this statement is not correct you must amend it as needed.  Please include this amended Role of Funder statement in your cover letter; we will change the online submission form on your behalf. | We have amended the cover letter accordingly. |
| Please note that funding information should not appear in the Acknowledgments section or other areas of your manuscript. We will only publish funding information present in the Funding Statement section of the online submission form. Please remove any funding-related text from the manuscript. | We have removed the funding section from the manuscript accordingly. |
| We note that you have indicated that there are restrictions to data sharing for this study. For studies involving human research participant data or other sensitive data, we encourage authors to share de-identified or anonymized data. However, when data cannot be publicly shared for ethical reasons, we allow authors to make their data sets available upon request. For information on unacceptable data access restrictions, please see [http://journals.plos.org/plosone/s/data-availability#loc-unacceptable-data-access-restrictions](http://url.uk.m.mimecastprotect.com/s/SSokCmy54uqq1WntQtQhR4LKD?domain=journals.plos.org). | We inadvertently ticked this box because we had not yet uploaded the data to a secure server.  We have now uploaded the raw data to a secure server, and the cleaned deidentified data are uploaded and available on request via (DataCompass) as per our institutional requirements. https://datacompass.lshtm.ac.uk/ |
| Before we proceed with your manuscript, please address the following prompts:  If there are ethical or legal restrictions on sharing a de-identified data set, please explain them in detail (e.g., data contain potentially identifying or sensitive patient information, data are owned by a third-party organization, etc.) and who has imposed them (e.g., a Research Ethics Committee or Institutional Review Board, etc.). Please also provide contact information for a data access committee, ethics committee, or other institutional body to which data requests may be sent.  b) If there are no restrictions, please upload the minimal anonymized data set necessary to replicate your study findings to a stable, public repository and provide us with the relevant URLs, DOIs, or accession numbers. Please see [http://www.bmj.com/content/340/bmj.c181.long](http://url.uk.m.mimecastprotect.com/s/zkGoCnO14cVVlmosEuohJKHyp?domain=bmj.com) for guidelines on how to de-identify and prepare clinical data for publication. For a list of recommended repositories, please see [https://journals.plos.org/plosone/s/recommended-repositories](https://url.uk.m.mimecastprotect.com/s/D1pwCoy14ummlvziWCrhpxmV9?domain=journals.plos.org). You also have the option of uploading the data as Supporting Information files, but we would recommend depositing data directly to a data repository if possible.  Please update your Data Availability statement in the submission form accordingly. | See previous response. |
| When completing the data availability statement of the submission form, you indicated that you will make your data available on acceptance. We strongly recommend all authors decide on a data sharing plan before acceptance, as the process can be lengthy and hold up publication timelines. Please note that, though access restrictions are acceptable now, your entire data will need to be made freely accessible if your manuscript is accepted for publication. This policy applies to all data except where public deposition would breach compliance with the protocol approved by your research ethics board. If you are unable to adhere to our open data policy, please kindly revise your statement to explain your reasoning and we will seek the editor's input on an exemption. Please be assured that, once you have provided your new statement, the assessment of your exemption will not hold up the peer review process. | See previous response. Data are available on request via Data Compass |
| One of the noted authors is a group or consortium: Delphi panel on EmONC signal functions and levels of care. In addition to naming the author group, please list the individual authors and affiliations within this group in the acknowledgments section of your manuscript. Please also indicate clearly a lead author for this group along with a contact email address. | We had already listed all of the individuals in the acknowledgements section. This is a large list. We have added the affiliations as we have them at the time, but are awaiting their confirmation to be listed as authors for the updated version of the manuscript. There is no lead author for this group. |
| We note that Figure 4 in your submission contain map images which may be copyrighted. All PLOS content is published under the Creative Commons Attribution License (CC BY 4.0), which means that the manuscript, images, and Supporting Information files will be freely available online, and any third party is permitted to access, download, copy, distribute, and use these materials in any way, even commercially, with proper attribution. For these reasons, we cannot publish previously copyrighted maps or satellite images created using proprietary data, such as Google software (Google Maps, Street View, and Earth). For more information, see our copyright guidelines: [http://journals.plos.org/plosone/s/licenses-and-copyright](http://url.uk.m.mimecastprotect.com/s/XugBCp215iZZQA8ukFmhGL4vp?domain=journals.plos.org). | We created that map uniquely based on our data using: [https://www.mapchart.net](https://url.uk.m.mimecastprotect.com/s/YdSRCwr14SlvEE9fVfriJn6tN?domain=mapchart.net) , and: [https://www.mapchart.net/world.html](https://url.uk.m.mimecastprotect.com/s/_s62Cx1g4cEL55wHvhviyrEHv?domain=mapchart.net). Created maps are licensed under/compatible with the [*Creative Commons Attribution-ShareAlike 4.0 International License*](http://url.uk.m.mimecastprotect.com/s/PfHvCyXjgiO7PPkHQiOixItZC?domain=creativecommons.org) |
| We require you to either (1) present written permission from the copyright holder to publish these figures specifically under the CC BY 4.0 license, or (2) remove the figures from your submission:  1) You may seek permission from the original copyright holder of Figure 4 to publish the content specifically under the CC BY 4.0 license.  We recommend that you contact the original copyright holder with the Content Permission Form ([http://journals.plos.org/plosone/s/file?id=7c09/content-permission-form.pdf](http://url.uk.m.mimecastprotect.com/s/KF9qCqZ14tvvLX5UMHLhEdCXi?domain=journals.plos.org)) and the following text:  “I request permission for the open-access journal PLOS ONE to publish XXX under the Creative Commons Attribution License (CCAL) CC BY 4.0 ([http://creativecommons.org/licenses/by/4.0/](http://url.uk.m.mimecastprotect.com/s/RP6aCr914SOOw2vtxIkh48Na-?domain=creativecommons.org)). Please be aware that this license allows unrestricted use and distribution, even commercially, by third parties. Please reply and provide explicit written permission to publish XXX under a CC BY license and complete the attached form.” | We have written to MapChart and they have signed the Content Permission Form, which we have included with the submission. |
| Please upload the completed Content Permission Form or other proof of granted permissions as an ""Other"" file with your submission. | As above. |
| In the figure caption of the copyrighted figure, please include the following text: “Reprinted from [ref] under a CC BY license, with permission from [name of publisher], original copyright [original copyright year].”    If you are unable to obtain permission from the original copyright holder to publish these figures under the CC BY 4.0 license or if the copyright holder’s requirements are incompatible with the CC BY 4.0 license, please either i) remove the figure or ii) supply a replacement figure that complies with the CC BY 4.0 license. Please check copyright information on all replacement figures and update the figure caption with source information. If applicable, please specify in the figure caption text when a figure is similar but not identical to the original image and is therefore for illustrative purposes only.    The following resources for replacing copyrighted map figures may be helpful:    USGS National Map Viewer (public domain): [http://viewer.nationalmap.gov/viewer/](http://url.uk.m.mimecastprotect.com/s/d3-_CvZ14tZZOA6uWSEhQbzaP?domain=viewer.nationalmap.gov)  The Gateway to Astronaut Photography of Earth (public domain): [http://eol.jsc.nasa.gov/sseop/clickmap/](http://url.uk.m.mimecastprotect.com/s/rwSJCwr14Snn0y1s4TRhJxOpU?domain=eol.jsc.nasa.gov)  Maps at the CIA (public domain): [https://www.cia.gov/library/publications/the-world-factbook/index.html](https://url.uk.m.mimecastprotect.com/s/hNJoCx1g4cjjmxAC4UPhy6hQk?domain=cia.gov) and [https://www.cia.gov/library/publications/cia-maps-publications/index.html](https://url.uk.m.mimecastprotect.com/s/tBEOCyXjgi336LMtKcLhxzOp1?domain=cia.gov)  NASA Earth Observatory (public domain): [http://earthobservatory.nasa.gov/](http://url.uk.m.mimecastprotect.com/s/DznoCzXkjizz842tNfQh9kQcX?domain=earthobservatory.nasa.gov)  Landsat: [http://landsat.visibleearth.nasa.gov/](http://url.uk.m.mimecastprotect.com/s/5TsJCA1pkc55rEWfqh2hGgsLy?domain=landsat.visibleearth.nasa.gov)  USGS EROS (Earth Resources Observatory and Science (EROS) Center) (public domain): [http://eros.usgs.gov/#](http://url.uk.m.mimecastprotect.com/s/Y-dVCB6q0UNNP84COiXh2qi-2?domain=eros.usgs.gov)  Natural Earth (public domain): [http://www.naturalearthdata.com/](http://url.uk.m.mimecastprotect.com/s/HWogCD8vnCZZj3VuqskhjYtx4?domain=naturalearthdata.com) | We have added this statement |
| Please include captions for your Supporting Information files at the end of your manuscript, and update any in-text citations to match accordingly. Please see our Supporting Information guidelines for more information: [http://journals.plos.org/plosone/s/supporting-information](http://url.uk.m.mimecastprotect.com/s/fGorCExwoHjj1pDC2t8h7VSMt?domain=journals.plos.org) | I have added supporting information file captions to the end of the manuscript. |
| Please review your reference list to ensure that it is complete and correct. If you have cited papers that have been retracted, please include the rationale for doing so in the manuscript text, or remove these references and replace them with relevant current references. Any changes to the reference list should be mentioned in the rebuttal letter that accompanies your revised manuscript. If you need to cite a retracted article, indicate the article’s retracted status in the References list and also include a citation and full reference for the retraction notice. | We have reviewed the reference list and do not cite any retracted papers |
| **Reviewer #1 Sanjoy Kumer Dey** |  |
| It is a excellent work which will help in maternal and newborn care in LMIC. Hence I have some observation mentioned below | Thank you for your peer review and helpful comments. |
| Title : Building consensus on revised Emergency Obstetric and Newborn Care (EmONC) signal functions and levels of care: A Delphi process and results : As Delphi process is a method of coming to a consensus, the title can be made short and more catchy | We appreciate this suggestion. Our new title is: A Delphi process to build consensus on revised Emergency Obstetric and Newborn Care (EmONC) signal functions and levels of care |
| Abstract : Result part better to emphasized more. | We have included specification of the number of signal functions to emphasise the results. |
| Result : Instead of terms like some/ many, these information can be expressed in percentage to quantify it | This is primarily a qualitative analysis. Wherever possible and appropriate, we have presented percentages to quantify the number of participants. For the qualitative thematic analysis, this is not always appropriate for all of the results. For these, we present exemplary quotes and an indication of where the balance of opinion lay (using some, many, most etc). |
| Discussion: New signal function proposed for both maternal and newborn health better to include in discussion section for better understanding of reader. Advantage part can be made short as there are some repeated information Limitation also can be make specific | The new signal functions are included in Figures 10a and 10b and referenced in the discussion as suggested. We have also added a final figure to show the final signal functions figure as it appears on the official website www.emonc.org, which will be the best place for people to view the revised set of signal functions and where the revised framework is also described alongside the accompanying indicators. We have shortened the advantages and limitations section so that it is more specific and avoids repetition. |
| **Reviewer #2: Anonymous** |  |
| Thank you for the opportunity to review this paper. It represents a significant contribution to the literature, and I believe it should be published and its publication highlighted by the journal. I have some comments on the study design and conclusions and suggestions to address before publication. | Thank you for these thoughtful and constructive comments. |
| The study design suited the objectives outlined by the authors. Signal functions have been in place for several decades and during that time have undergone minimal revision. Their utility has been debated and, as the overarching project suggests, re-visioning was necessary. Involving multiple stakeholders with varying expertise and experience, was also critical. While the drop out rate seems relatively high, the authors point out that a completion rate of 53% for a Delphi process such as this is not atypical. Might it have been improved and if so, how? | In all rounds, reminder emails were sent to all participants, and in some cases the deadline extended where reasonable. We have added this to the discussion of advantages (line 480-481) Due to the fixed timeline for the second technical workshop, some participants were still on summer leave. Carrying out this round later in the year may have increased participation, but would not necessarily have changed the overall findings. Largely, the biggest factor affecting participation was the post pandemic landscape and people struggling with busy schedules and more pressing priorities, especially those working in clinical settings and/or in the field. |
| Difficulties were faced in recruiting a suitably wide range of participants. Several groups were overrepresented, for example those who identified as academic researchers and program managers. The authors point out that participation from front line care providers was challenging. What impact might this have had on the results? | Previous studies ([Moxon et al](https://journals.plos.org/plosone/article?id=10.1371/journal.pone.0218748), 2019) have shown that in these types of surveys, frontline providers and those with more experience working in lower income settings were more likely to assign a higher level of care for more complex interventions. With this in mind, technical discussions were aimed at striking a balance between aspirational and realistic signal functions for different levels of care for the revised EmONC framework.  It was always likely that this type of study would be over represented by academic researchers and programme managers – more desk-based roles. Front line providers were essential to include in the overall project and part of the reason that the Delphi was embedded in a wider process, with human centred design principles at the core. Front line providers were involved in all technical discussions so their voice was incorporated into the final decision making processes and the creation of materials for each round. We have inserted more interpretation in line 515-518. |
| In addition, while the three rounds are clearly described and the iterative process was clear, there appear to have been late additions to the list of signal functions that are not well described. Breast feeding for example, makes a final appearance on the list with the statement that it was added by the newborn expert group to be consistent with WHO guidelines. This is not addressed in the text. Did it meet criteria for a signal function or would it actually fall within routine care? There is some ambiguity about whether the signal functions cover "routine and non-clinical care dimensions." However, the paper suggests a consensus was reached on differentiating emergency from routine care. Further justification is needed for including breastfeeding as a signal function. | Thank you for this comment. At the start of the process, strong arguments were made for including elements of routine care, but the consensus was that the focus of the final framework would be on emergency care. Breastfeeding was not considered within the Delphi because it was seen as routine not emergency or complication care There were lengthy discussions on breastfeeding, and ultimately it was included due to a strong recommendation from WHO, and a desire to keep them on board to endorse the revised signal functions. In the text we do explain this in the challenges section, and have added wording to make it clearer (line 493). |
| At the outset it was stated that in order to achieve consensus, 85% agreement would be sought. Only 14/26 or 53% actually achieved this level of consensus. Why was this the case and was it anticipated? Although it is peripherally addressed in the paper, more attention should be given to the lack of consensus and why this might be. | We intended to include all functions that reached 85%, but did not expect all included functions to reach this level. We have clarified the text (line 425-427) to make this clearer. As the reviewer points out, for over half of the signal functions we had ≥85% consensus from Delphi participants, for the remainder, we had built in technical workshops to discuss areas with lack of consensus. Figures 10a and 10b show which signal functions did not achieve consensus, and a summary of reasons. Some of the lack of consensus was around the level, e.g. what level should assisted vaginal delivery be at. Other reasons stemmed from differences in stages of implementation in different countries (e.g. Retinopathy of prematurity for newborns). We have added some wording on lines 426-428 |
| There is mention of transparency, but what were the feelings of participants regarding the lack of consensus and was a plan in place from the beginning to address this (beyond the technical workshops)? Was that fully explained to participants? | This was explained to participants. All participants received a participant information sheet and were informed that the study was looking “*to solicit knowledge and a level of consensus from experts in maternal and newborn health…”* They were also informed that the project was committed to “*fully integrating newborn care signal functions as part of the revision”.* They were also informed in this information sheet that “*some may find the consensus process challenging and whilst we will do whatever we can to manage diverse opinions, there will always be some compromise…”.* The participant information is included in the supplementary material alongside other material shared for R1. How fully this was understood by participants in not clear to us.  All participants in the technical workshops were given the opportunity to contribute to the Delphi study, but there was not sufficient funding available to involve all Delphi participants, which is a limitation.  All participants have been sent the manuscript draft with an accompanying email describing the next steps until WHO formally endorses the signal functions. They will be given the opportunity to keep their name as a co-author on the publication or to have it removed. |
| In round 2 participants were presented with a scenario that was situated in a Comprehensive EmONC setting. Was consideration given to having participants think in a similar fashion about a first line setting, and if not, why not? | We chose to focus on a comprehensive EmONC setting as we anticipated, particularly from the newborn side that this was the level that would require the greatest number of new signal functions and the decision was strategic given the focus on scaling up level 2 newborn care. We have included a few lines (522-524) to acknowledge that if resources had allowed, an additional round could have looked at BEmONC, but instead we wanted the final round to consolidate all three levels. |
| The transfer signal function, one which I believe was felt to be an important addition, is hardly discussed at all. What was the consensus around this signal function and why is it not included In figures 9, 10a or 10b? | The transfer/referral signal function is included in figure 10a (with a note added that it should be collected with newborn and obstetric signal functions). Round 1 of the Delphi showed strong support for including a signal function or clear measure of referral in the revised framework and this signal function was proposed and elaborated by a group of technical experts working on referral. |
| I would like to address the signal functions themselves and the figures used to illustrate them. Figure 10a outlines the list of maternal SF’s while 10b lists the newborn SF’s There appear to be 12 in total for maternal, not including the transfer SF and 14 newborn. It is possible that I have miscounted however 10b suggests that there are 2 at the intensive level, 7 at comprehensive level and 5 at basic level (total of 14 not 13). This would be a total of 27 if one includes the referral SF. I suspect that if I am confused others will be as well! | There are 25 signal functions in total.  Figure 10a - obstetric. There are 11 obstetric signal functions, not including the referral signal function.  Figure 10b – neonatal. There are 13 newborn signal functions, but oxygen appeared twice in the submitted figure. The figure has been amended and additional text added to describe that it is measured differently at basic and comprehensive level. Initially, this was going to be considered a separate signal function but it was agreed that this is confusing. This has been amended accordingly to better show the 25 signal functions.  We have amended this in the figure to better reflect how it will be described/presented in the final WHO documents and the final signal functions can be viewed in figure 11. |
| In addition it is unclear to me if the term “basic” is being used or “First level” . Figure 7 uses “first level” while figures 10 a and b use “basic”. Have we settled on that terminology? | You are correct, this should have been amended in the final figure. Basic is the correct terminology. |
| Figure 1 is helpful historically Figure 2 does not add to the paper. While it is important that the Delphi process be shown to be embedded in the project as a whole, the figure does not help to visualize this. What might be more helpful would be to indicate where the Delphi process and re-visioning of the signal functions is situated within the framing of the signal function (clinical, health systems and human rights). | Thank you for this comment, while we appreciate you do not feel this figure adds to the paper, we have had a lot of feedback from co-authors and presentations saying they find the figure helpful. Therefore we would like to retain the figure. |
| My comment above highlights the fact that another graphic similar to Figure 7 with the updated final list would be helpful. | We have now included a link to the www.emonc.org , the official website of the Re-visioning EmONC project where the final EmONC signal functions and framework are described and presented in detail. We were conscious of having a lot of figures, but if the editors permit, we would like to add the diagram from emonc.org to this paper as an additional figure as an additional, final figure 11. |
| I would also suggest that figure 9 be revised to more clearly indicate which SF’s reached the threshold of consensus. Percentages are included but a different colour or cross hatching might help to visualize how many required more discussion and consensus building. | We have adapted the figure to show the signal functions in a different colour which did not reach >85% consensus. The length of the bar was also designed to show the percentage. |
| Finally, what are the next steps in order to operationalize these changes? This should be part of the concluding remarks. How do we develop measurement processes to track and how will countries move forward with these changes? Clearly these issues cannot be addressed fully here, but should be introduced as follow up projects. | We have added some additional wording to the concluding remarks. |
